# Heterovalent, Homometallic Rings: Synthesis, Optical, and EPR Studies of {Ti^IV^_7_Ti^III^} Complexes

**DOI:** 10.1002/chem.71451

**Published:** 2026-07-18

**Authors:** Selena J. Lockyer, Lubomir Loci, Yingzhao Ma, Matthew P. Stevens, Grigore A. Timco, George F. S. Whitehead, Richard E. P. Winpenny, Alice M. Bowen, Eric J. L. McInnes

**Affiliations:** ^1^ Department of Chemistry and Photon Science Institute The University of Manchester Manchester England

**Keywords:** electron spin relaxation, EPR, metallacycles, mixed valent, titanium oxo clusters

## Abstract

We report a new class of heterovalent, homometallic ring complexes, with {Ti^IV^_7_Ti^III^} cores: [py‐CH_2_NH_2_Et][Ti_7_TiO_8_(O_2_CR)_16_] (R = ^t^Bu and cyclopropyl). The compounds are made from [Ti(O^i^Pr)_4_] reacted with carboxylic acid and py‐CH_2_NHEt. We also show that {Ti^IV^_7_Ti^III^} rings can be generated by photoreduction of {Ti^IV^_8_} precursors in the presence of a suitable sacrificial electron donor, of relevance to photocatalysts. UV–vis/NIR spectroscopy of the {Ti^IV^_7_Ti^III^} rings reveal Robin and Day Class II mixed‐valent behavior at room temperature, while EPR spectroscopy is consistent with a localized 3d^1^ (*s* = 1/2) configuration, with C_2_ local symmetry, at low temperatures. We compare the EPR behavior of these new rings with the isostructural heterometallic {Cr^III^_7_Ni^II^} rings [py‐CH_2_NH_2_Et][Cr_7_NiF_8_(O_2_CR)_16_], which also have *s* = ½ ground states, due to strong intramolecular antiferromagnetic exchange interactions. We demonstrate that the electron spin lattice relaxation time constants (*T*_1_) for {Ti^IV^_7_Ti^III^} are over two orders of magnitude longer at low temperatures than for the {Cr_7_Ni} equivalents, and that phase memory time constants (*T*_m_) are maintained to much higher temperatures.

## Graphical abstract

A new class of heterovalent, homometallic complexes is reported, based on cyclic {Ti^IV^_7_Ti^III^} cores with oxide and carboxylate bridges between nearest neighbors. The clusters show Class II mixed‐valent behavior at room temperature, but are valence localized at low temperature. The *s* = ½ ground state, and much longer electron spin relaxation time constants, are contrasted with those of isostructural, exchange‐coupled {Cr^III^_7_Ni^II^} rings.

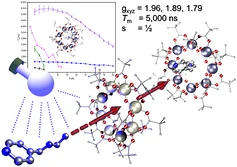

## 1 Introduction

Metallacycles are of interest in areas including supramolecular chemistry and molecular magnetism. In the former they have formed structural components of for example complex knots, catenanes, and rotaxanes [1, 2, 3, 4]. In the latter, they were initially studied as finite models of low‐dimensional magnets, but it rapidly became clear that their magnetic properties were important in their own right. Much of the original work was on homometallic and homovalent complexes, for example Lippard's [Fe^III^_10_(OMe)_20_(O_2_CCH_2_Cl)_10_] [5] and Gerbeleu's [Cr^III^_8_F_8_(O_2_C^t^Bu)_16_] [6, 7]. Further interest came with predictions of interesting behavior that could result from symmetry breaking [8], through odd numbers of metal ions or through a dopant site in the ring. We have studied such systems of general formula [cation][Cr^III^_7_M^II^F_8_(O_2_CR)_16_] [9, 10]. The {Cr^III^_7_Ni^II^} family has attracted particular interest because strong antiferromagnetic coupling between the Cr^III^ (*s* = 3/2) and Ni^II^ (*s* = 1) ions leads to a well‐isolated *s* = ½ ground state, and have been proposed as potential qubits in quantum information processing [11, 12]. These do not have the coherence times achievable with simpler metal–organic [13, 14, 15, 16] or organometallic compounds [17, 18], but can be linked into supramolecular arrays in multiple ways and with great synthetic control [1, 2, 19, 20, 21, 22, 23]. As it is straightforward to link qubits based on these polymetallic complexes it would be valuable to improve the coherence times. Other groups are making multi‐qubit molecules, for example based on linked radicals [24], heterometallic lanthanide complexes [25, 26], and on linked porphyrins [27, 28].

We reasoned that reducing the number of unpaired electrons in the ring should influence relaxation, and therefore we targeted {Ti^IV^_7_Ti^III^} rings which would have a single unpaired electron (Ti^IV^, 3d^0^; Ti^III^, 3d^1^) hence *s* = ½, to contrast with {Cr^III^_7_Ni^II^} rings (Cr^III^, 3d^3^; Ni^II^, 3d^8^) which contain twenty‐three unpaired electrons. A further impetus for such compounds is that {Ti^IV^_8_O_8_} rings are the inorganic nodes in an important class of metal–organic framework (MOF) materials, the parent MOF being MIL‐125 which has the formula Ti_8_O_8_(OH)_4_(O_2_C‐C_6_H_4_‐CO_2_)_12_ [29]. MIL‐125 is an efficient photocatalyst which involves the generation of Ti(III) sites on UV–vis excitation; such materials give an avenue to combine the porosity of MOFs with the heterogeneous photocatalytic properties of TiO_2_. Molecular analogues would have potential application in homogeneous catalysis.

## 2 Results and Discussion

Here we compare four single ring compounds: [Cation][Cr^III^_7_Ni^II^F_8_(O_2_C^t^Bu)_16_] **1**, [Cation][Cr^III^_7_Ni^II^F_8_(CPC)_16_] **2**, [Cation][Ti^IV^_7_Ti^III^O_8_(O_2_C^t^Bu)_16_] **3,** and [Cation][Ti^IV^_7_Ti^III^O_8_(CPC)_16_] **4**, where Cation = (py‐CH_2_NH_2_Et)^+^ (py = pyridyl) and CPC = cyclopropyl‐carboxylate (Figure 1). Complexes **1** and **2** have been reported [21, 23]. The two different carboxylates are chosen to compare the effects of the ligands and of the metal core on the electron spin relaxation properties. We have previously reported heterometallic rings (R_2_NH_2_)[Ti^IV^_7_M^III^O_8_(O_2_C^t^Bu)_16_] (M = Cr, Fe, Al, Ga, and In) [30] based on the observation that the {Ti^IV^O(O_2_CR)_2_} unit is isostructural with, and has the same charge as, {Cr^III^F(O_2_CR)_2_}. A parent homovalent {Ti^IV^_8_} ring is known, namely [Ti_8_O_8_(O_2_CC_6_F_5_)_16_] [31]. Complexes **3** and **4** were made by related reactions as {Ti^IV^_7_M^III^}, but without the heterometal source: [Ti(^i^OPr)_4_] is added to a mixture of carboxylic acid and py‐CH_2_NHEt under heat, in an inert atmosphere to give blue products in 30%–50% yield (see Supporting Information for details). The blue products are only seen with this specific amine, which must also be the reducing agent (see below). Related alkylaminopyridines have been studied in the context of dye‐sensitized solar cells based on TiO_2_ [32].

**FIGURE 1:**
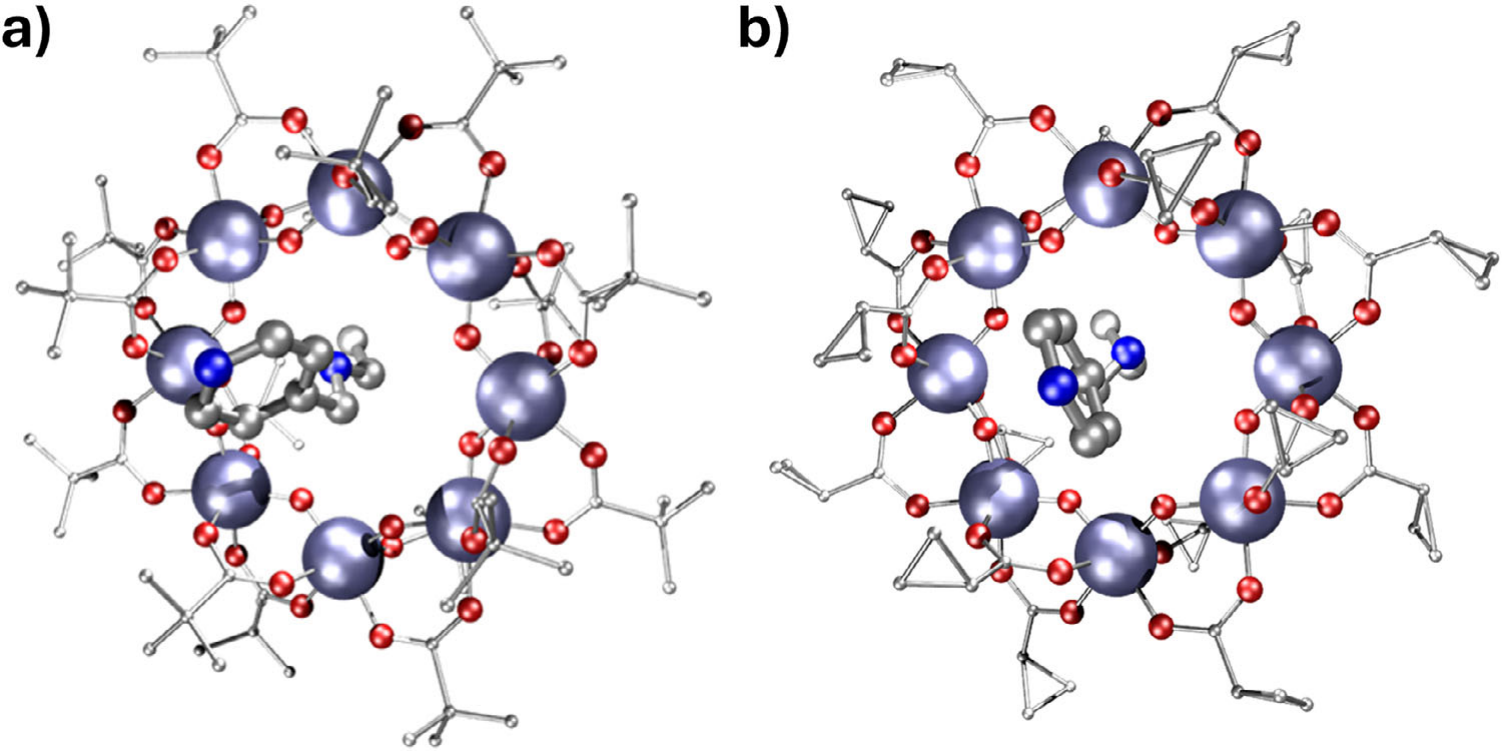
The structures in the crystal of (a) **3** and (b) **4**. Color scheme: Ti, dark blue; O, red; N, blue; C, grey. H‐atoms excluded for clarity.

In each of **1**‐**4** the structures [33] (see Table S1 for crystallography details) contain eight metal sites forming an octagon, with each edge of the octagon bridged by two carboxylates and a monodentate ligand, which is fluoride in **1** and **2** and oxide in **3** and **4** (Figures 1 and S1). In **1** and **2** the divalent metal, and in **3** and **4** the trivalent metal ion, is disordered about the octagon of metals. The Ti‐oxide distances in **3** and **4** are 0.1 Å shorter than the M‐F distances in **1** and **2** (Table S2), consistent with the higher charges. A charge balancing cation is found at the centre of the ring; here it is a protonated secondary ammonium ion with a pyridyl group on one of the side‐chains. We have previously used this thread to bond to other metal complexes via the terminal pyridyl [34]. The bond lengths and angles at the metal sites are given in Table S2 and are unremarkable.

Complexes **1** and **2** are green in colour due to the Cr(III) dd transitions [35]. Complexes **3** and **4** are blue. Solution state UV–vis/NIR spectra show this is due to dominant, very broad transitions at 𝜐_max_ = 13.5 and 13.9 x 10^3^ cm^−1^ in **3** and **4**, respectively, (Figure 2). These bands have extinction coefficients ($\varepsilon_{\max}$) of 230 and 680 M^−1^ cm^−1^, respectively, with FWHMs of ∆𝜐_1/2_ ≈ 10 x 10^3^ cm^−1^ (this is an overestimate if more than one transition is present). Two much weaker and narrower features are observed at ca. 19.2 and 23.8 x 10^3^ cm^−1^ in both complexes ($\varepsilon_{\max}$ ca. 10^0^ ‐ 10^1^ M^−1^ cm^−1^). The latter are consistent with dd transitions associated with the 3d^1^ Ti(III) ion. The former are not present in parent all‐Ti(IV) compounds, and the FWHM, extinction coefficients and absorption maxima are consistent with inter‐valence charge transfer (IVCT) transitions: these parameters combine to give a weak electronic coupling parameter $H_{ab}={\left(4.2\times 10^{-4}\varepsilon_{\max}\upsilon_{\max}\Delta \upsilon_{1/2}\right)}^{1/2}/r$ where *r* is the Ti(IV)…Ti(III) separation in Å: here *H*_ab_ is ca. 10% of 𝜐_max_ [36, 37]. The presence of both dd and IVCT confirms these as Class II mixed‐valence compounds in the Robin and Day classification [38]. Similar IVCT transitions have been observed in MIL‐125 derivatives [29, 39] and Ti alkoxide clusters [40] after UV–vis irradiation in the presence of a sacrificial electron donor (e.g., during substrate photooxidation reactions). It is not clear how many sites in the octametallic ring the unpaired electron is delocalized over, nor how this varies with temperature. It may be of interest to study this in a finite, molecular system with relevance to polaron hopping in photoexcitation of extended lattices.

**FIGURE 2:**
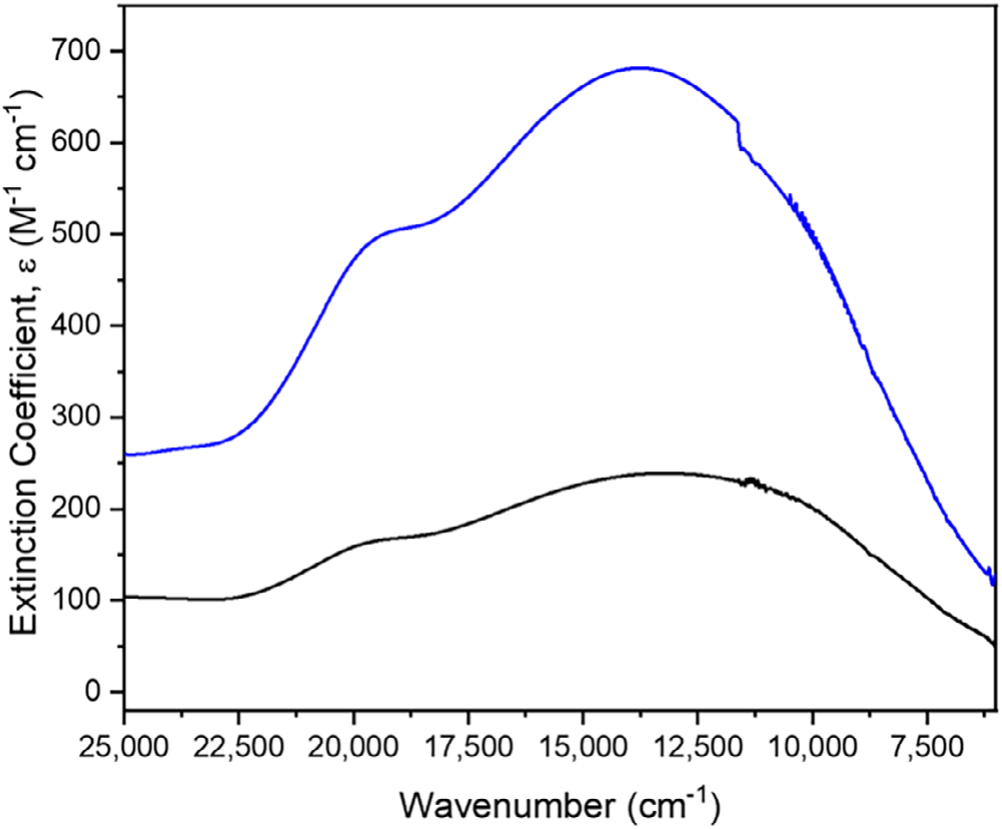
UV–vis/NIR spectra of **3** in toluene solution (black trace) and **4** in CH_2_Cl_2_ solution (blue), at room temperature.

As seen for other {Cr^III^_7_Ni^II^} rings, the EPR spectra of **1** and **2** at low temperature are dominated by the *s* = ½ ground state resonance at ca. *g* = 1.8 that arises from strong intramolecular antiferromagnetic coupling between nearest neighbours [9, 10, 41]. Above ca. 20 K, a broad resonance grows at around *g* = 1.98 due to population of further and numerous excited states [23].

For **3** and **4**, well‐resolved rhombic *s* = ½ continuous wave (cw) EPR spectra are observed at X‐ and Q‐band in the solution and powder states (Figures 3, S3 and S4); simulation of the solid‐state spectra (Figures S5, S6) gives *g* = 1.955, 1.880, and 1.770 for **3** and *g* = 1.963, 1.888, and 1.793 for **4**. The observed linewidths are around half those for **1** (Table S3). The *g*‐values are all below the free electron value (*g*_e_ = 2.0023) hence consistent with isolated 3d^1^ Ti(III) sites, and the narrowness of the spectra is consistent with valence‐localized centres. At X‐band, and in solution and powder, there is a noticeable broadening introduced above ca. 100 K (Figures S7–S11), becoming pseudo‐isotropic (and very weak) above ca. 200 K. Little signal is observed at higher temperatures. The high‐temperature behavior is consistent with the onset of rapid relaxation via electron hopping between the Ti sites in the ring, as evidenced by the IVCT band in the room temperature UV–vis/NIR spectra. Similar variable temperature behavior is observed for Ti(III) sites in reduced TiO_2_ [42].

**FIGURE 3:**
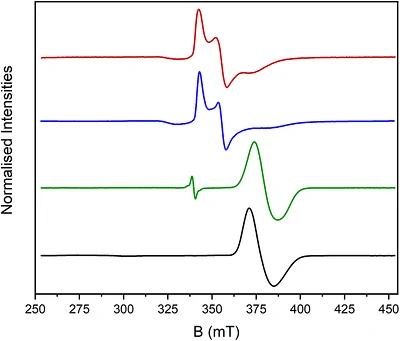
The cw EPR spectra of **1** ‐ **4** (black to purple lines, respectively) at X‐band and 5 K measured as a 2 mM solution in toluene for **1** and **3**, and in CH_2_Cl_2_ for **2** and **4**.

The low temperature *g*‐values are much more anisotropic than observed for Ti(III) in regular lattice sites of reduced TiO_2_ ($g_{⊥,∥}=$ 1.99, 1.96 in anatase; *g* = 1.97, 1.96, and 1.95 in rutile) [42]. However, they are rather close to those observed for Ti(III) in MgO (*g* = 1.95, 1.85, and 1.77) where the significant rhombic distortion was assigned to a single cation vacancy neighbouring the coordinated oxides in the local equatorial plane giving local C_2v_ symmetry (where the smallest *g*‐value defines the local z‐axis) [43]. In **3** and **4**, the [Ti^IV^_7_Ti^III^O_8_(O_2_CR)_16_]^−^ rings (*i.e*. neglecting the thread) have C_2_ symmetry where the C_2_ axis passes through the Ti(III) and the antipodal Ti(IV) ion. Hence, the local symmetry at Ti(III) is C_2_, but if we just consider the first coordination sphere of *cis*‐{TiO^oxide^_2_O^carboxylate^_4_} then we can approximate to C_2v_ (Figure 4). For convenience, we define the C_2_ axis as x, with z defining the O^carboxylate^‐Ti‐O^carboxylate^ direction that is near normal to the ring (in fact this vector is rotated ca. 15° from the normal to the best {Ti_7_Ti} plane), leaving y bisecting the equatorial O^carboxylate^‐Ti‐O^oxide^ angles hence near tangential to the rings (Figure 4). In this axis system the “*t*_2g_” and “*e*_g_” manifolds are ($d_{\mathrm{xz}},d_{\mathrm{yz}},d_{x^{2}-y^{2}}$) and ($d_{\mathrm{xy}},d_{z^{2}}$), respectively. The two *cis*‐oxides break the symmetry; in C_2v_ (or C_2_) the degeneracy is completely broken. If we assume the 2− charges of the oxides dominate then the $d_{yz}^{1}$ configuration is stabilized. In this case perturbation theory gives the following expressions for the *g*‐values:

$$g_{\mathrm{xx}}=g_{e}-\frac{2\lambda }{\delta_{x^{2}-y^{2}}}-\frac{6\lambda }{\delta_{z^{2}}}$$

$$g_{\mathrm{yy}}=g_{e}-\frac{2\lambda }{\delta_{\mathrm{xy}}}$$

$$g_{\mathrm{zz}}=g_{e}-\frac{2\lambda }{\delta_{\mathrm{xz}}}$$

where λ is the spin‐orbit coupling constant for Ti(III) (we take this as the free‐ion value of 154 cm^−1^), and $\delta_{i}$ is the energy gap between the $d_{\mathrm{yz}}$ SOMO and $d_{i}$ orbital. If we assign the experimental dd transitions at 19.2 and 23.8 x 10^3^ cm^−1^ as excitations from $d_{\mathrm{yz}}$ to the “*e*_g_” pair of $d_{z^{2}}$ and $d_{\mathrm{xy}}$, respectively, then we calculate *g*_yy_ = 1.99. Back‐calculating from the experimental *g*_xx_ and *g*_zz_ gives $\delta_{x^{2}-y^{2}}$ = 3.7 x 10^3^ cm^−1^ and $\delta_{\mathrm{xz}}$ = 1.4 x 10^3^ cm^−1^; these energies are consistent with the scheme in Figure 4 and would not be observable in the UV–vis/NIR spectrum.

**FIGURE 4:**
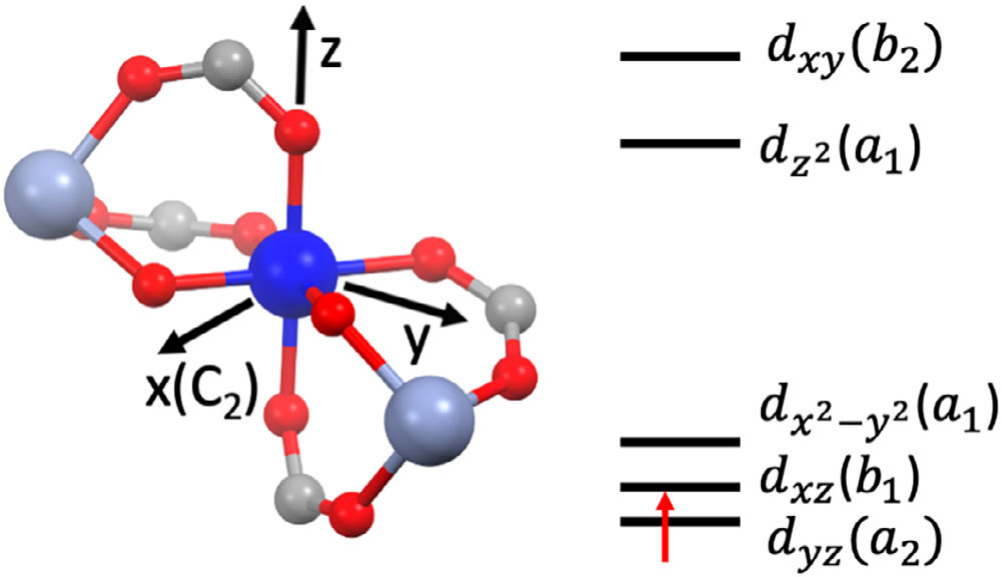
Left: local structure at the Ti(III) site, showing Ti(III) (blue) and two neighbouring Ti(IV) (dull blue) ions (R groups of carboxylates omitted for clarity), and axis scheme. Right: resulting crystal field splitting (C_2v_ representations in parentheses; C_2_ labels are the same omitting the subscript).

Echo‐detected field‐swept (EDFS) pulsed EPR spectra from dilute (0.2 mM) solutions of **1** ‐ **4** at 5 K are consistent with cw EPR spectra (Figure S12). The spin‐lattice relaxation (*T*_1_) and phase memory (*T*_m_) time constants were measured using established pulse sequences (see Figures S12–S26; Tables S4–S12). The two {Ti^IV^_7_Ti^III^} rings (**3** and **4**) have similar *T*_1_ behavior, with maximum values of 3.0 ± 0.2 ms at 4 K, decreasing to ca. 10 µs at 20 K (Figure 5b). At 4 K, these *T*_1_ values are over two orders of magnitude greater than those of the equivalent {Cr^III^_7_Ni^II^} rings **1** and **2** (for which echoes are only detectable below ca. 5 K; Table S10) [12, 23]. The much longer *T*_1_ for **3** and **4** is due to the localized d^1^ configuration of the Ti(III) ion at these temperatures. In contrast, although **1** and **2** have well‐isolated *s* = ½ ground states, they have a high density of magnetic states due to the exchange coupling between the seven Cr(III) and Ni(II) ions [first excited states (*s* = 3/2) at 10 and 8 cm^−1^, respectively] [23]. This provides an efficient mechanism for *T*_1_ relaxation [44]. As observed for **1** and **2**, there is relatively little difference in *T*_1_ between the CPC and pivalate complexes **3** and **4**.

**FIGURE 5:**
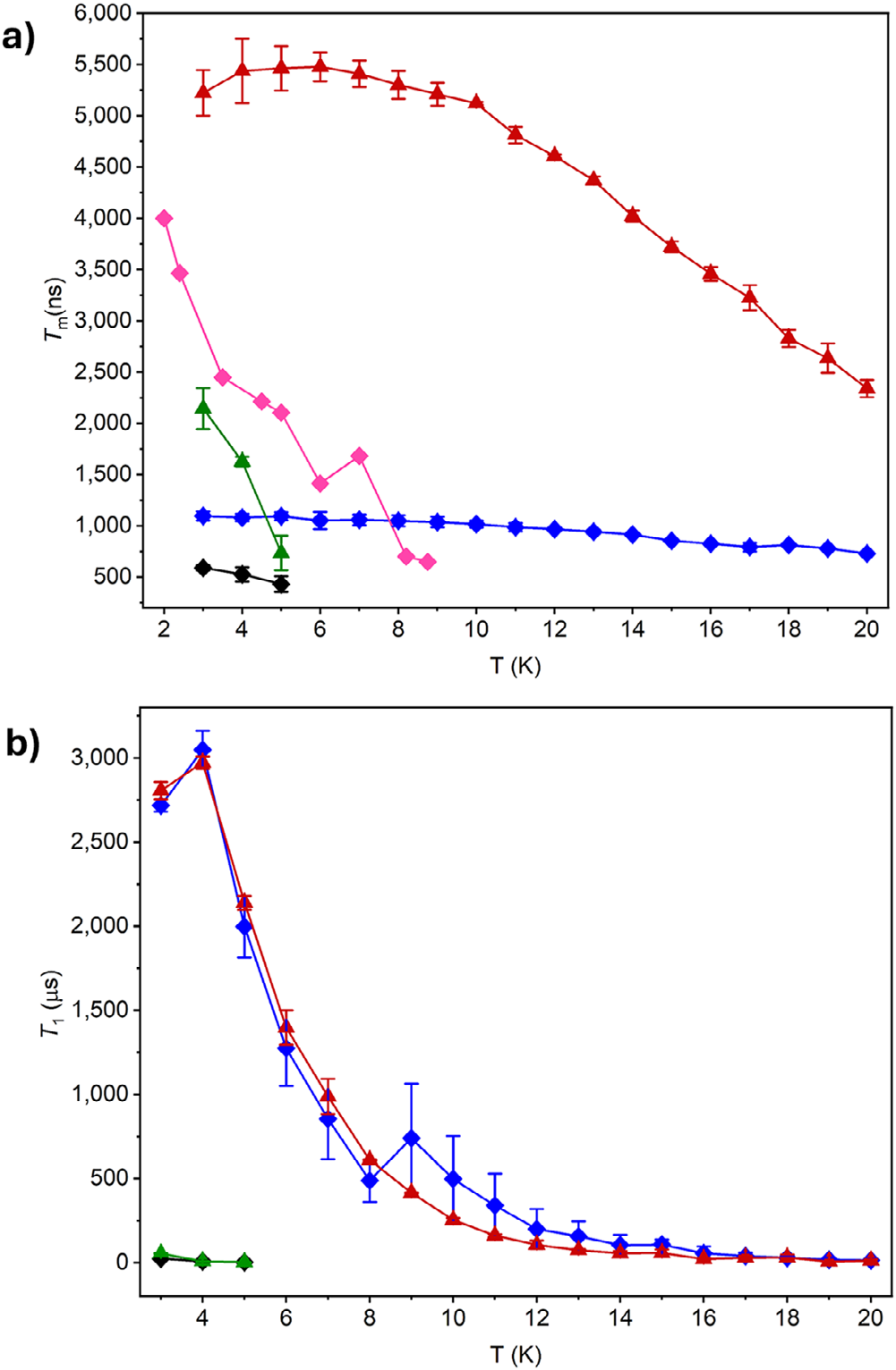
Temperature dependence of (a) *T_m_* and (b) *T_1_* for **1** (black diamonds), **2** (green triangles), **3** (blue diamonds), and **4** (red triangles). *T*_m_ data for perdeuterated [Et_2_NH_2_][Cr^III^_7_Ni^II^F_8_(O_2_C^t^Bu)_16_] shown for reference (pink diamonds) [46].

For a common carboxylate ligand, the {Ti^IV^_7_Ti^III^} rings have a longer *T*_m_ than {Cr^III^_7_Ni^II^} by a factor of ca. 2 at a temperature of 3 K, with **3** and **4** having *T*_m_ of 1.1 ± 0.05 and 5.2 ± 0.2 µs, respectively, (Figure 3a). (0.4 and 0.2 mM solutions give the same *T*_m_, hence intermolecular effects are not dominating.) In contrast to the *T*_1_ behavior, the use of CPC instead of pivalate has a large effect on *T*_m_, increasing *T*_m_ in **4** compared to **3** by a factor of ca. 5 at 3 K. The weak temperature dependence of *T*_m_ in **3** is indicative of dephasing dominated by nuclear spin diffusion [44], and this is partially quenched in **4** by having fewer ^1^H nuclei per carboxylate ligand (5 vs. 9 in CPC and pivalate, respectively,) [23]. In addition, use of CPC, which has no methyl groups, quenches dephasing induced by Me tunnelling rotation [45]. Measurements using the CPMG sequence give longer time constants for both **3** and **4** (*T*_CMPG_/*T*_m_ = 5.2 for **3**, 2.8 for **4**, at 3 K; Table S8), indicating that spectral diffusion also contributes to dephasing [44].

We have reported similar trends in *T*_m_ behavior for the {Cr^III^_7_Ni^II^} rings **1** and **2** [23]. However, for **2** the gain in *T*_m_ (cf. **1**) is only at the lowest temperatures with similar values observed at 5 K because *T*_m_ becomes limited by *T*_1_ induced spectral diffusion. In contrast, for **3** *T*_m_ only decreases from 1.1 to 0.7 µs between 3 and 20 K; that for **4** decreases by more but at 20 K is still larger than for **3** by a factor of 2.5. The persistence of *T*_m_ with increasing temperature is because of the much longer *T*_1_ over the temperature range. The effect of the metal core is illustrated by the fact that, even though relaxation at low temperature is dominated by nuclear spins, **3** has significantly longer *T*_m_ than a perdeuterated {Cr^III^_7_Ni^II^} complex (Figure 5) [46]. The longer *T*_m_ times of **3** and **4** can be exploited to perform more complex pulsed EPR experiments and to higher temperature. For example, in transient nutation experiments Rabi oscillations can be observed to much longer nutation times, and to much higher temperatures (we have measured up to 20 K) for compounds **3** and **4** than for **1** and **2** (Figures S31–S38). This study demonstrates that significant differences in relaxation behavior can be observed for polymetallic qubit candidates, achieved both by changing the ligand type and also by changing the electronic structure of the core.

Finally, given the importance of TiO_2_ and the {Ti_8_} based MOFs in photocatalysis, we examined whether {Ti^IV^_7_Ti^III^} rings could be formed by irradiation of {Ti^IV^_8_} rings. Irradiating a solution of [Ti^IV^_8_O_8_(O_2_C^t^Bu)_16_] **5**, in the presence of Et_2_NH and excess pivalic acid (providing a proton source to form Et_2_NH_2_^+^), with a Xe arc lamp under anaerobic conditions gives blue solutions which have similar EPR signals to **3** and **4** (Figure 6). The EPR signal intensity varies with both the **5**:amine ratio and the irradiation power, which confirms that a photochemical reduction takes place although the precise mechanism is not clear. Future work will examine this photochemical process including in the presence of substrates that can be reduced, as in the MOFs.

**FIGURE 6:**
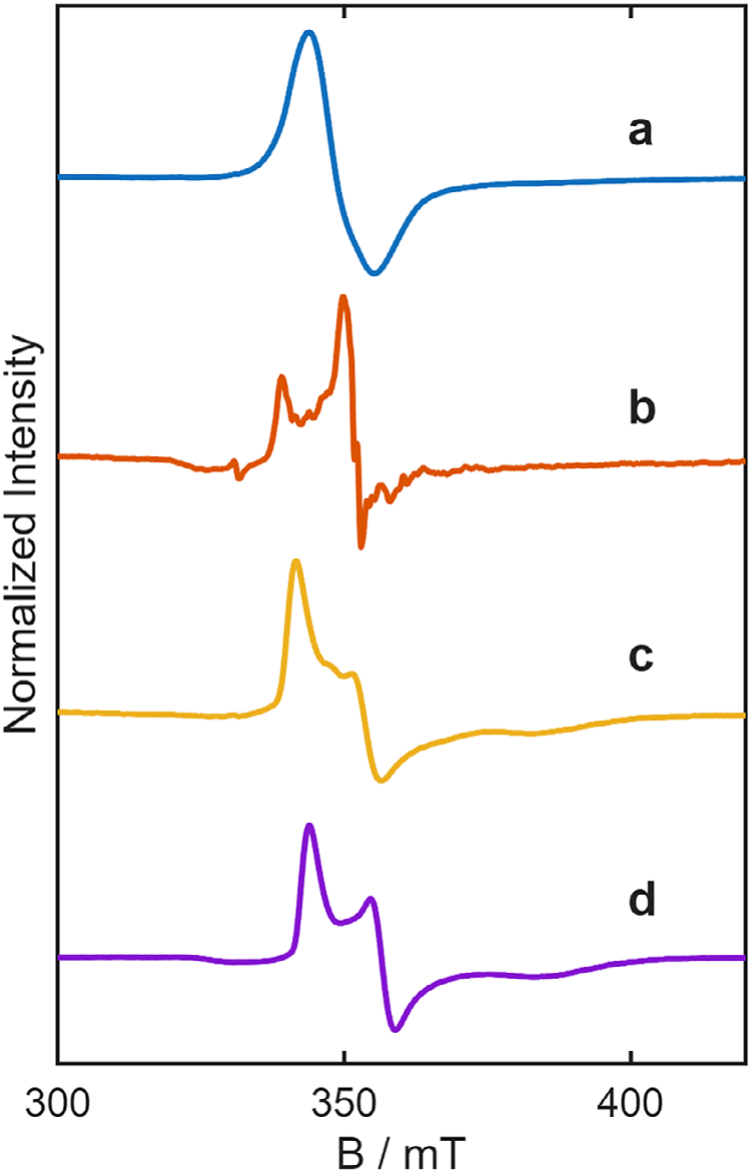
CW X‐band EPR spectra of {Ti^IV^_7_Ti^III^} rings (200 𝜇M toluene solution) produced by photochemistry. (a) 1:1 **5**:Et_2_NH at high power; (b) 1:9 **5**:Et_2_NH at low power; (c) 1:9 **5**:Et_2_NH at high power; (d) compound **3** for comparison.

## 3 Conclusion

In conclusion, a new class of homometallic, heterovalent rings based on the {Ti^IV^_7_Ti^III^} core can be prepared directly from monometallic Ti^IV^ precursors or photochemically from {Ti^IV^_8_} analogues. They have electron spin *s* = ½ ground states that are delocalized (Robin and Day class II) at room temperature but localized Ti^III^ at low temperatures, with much slower electron spin relaxation properties at low temperatures than their isostructural {Cr_7_Ni} analogues. Future work will seek to exploit these properties in molecular magnetism and photochemical applications.

## Conflicts of Interest

The authors declare no conflicts of interest.

## Supporting information




**Supporting file**: chem71451‐sup‐0001‐SuppMat.pdf.
All data further supporting this study is provided in the Supplementary Information (SI) file accompanying this paper. The authors have cited additional references within the Supporting Information [47, 48, 49, 50, 51, 52].

## Data Availability

The data that support the findings of this study are available on request from the corresponding author. The data are not publicly available due to privacy or ethical restrictions.

## References

[1] C.‐F. Lee , D. A. Leigh , R. G. Pritchard , et al., “Hybrid Organic–Inorganic Rotaxanes and Molecular Shuttles,” Nature 458 (2009): 314–318, 10.1038/nature07847. 19295605

[2] S. J. Lockyer , G. F. S. Whitehead , G. A. Timco , E. J. L. McInnes , and R. E. P. Winpenny , “Hybrid Inorganic‐Organic Rotaxanes and Related Compounds,” Chemistry—A European Journal 31 (2025): e202501067, 10.1002/chem.202501067. PMC12202853 40386965

[3] Y. J. Liu , C. Zheng , H. Xiao , et al., “Designed Synthesis of an Aluminum Molecular Ring Based Rotaxane and Polyrotaxane,” Angewandte Chemie International Edition 63 (2024): e202411576, 10.1002/anie.202411576. 38984566

[4] S.‐L. Huang , T. S. A. Hor , and G.‐X. Jin , “Metallacyclic Assembly of Interlocked Superstructures,” Coordination Chemistry Reviews 33 (2017): 1–26, 10.1016/j.ccr.2016.11.009.

[5] K. L. Taft , C. D. Delfs , G. C. Papaefthymiou , S. Foner , D. Gatteschi , and S. J. Lippard , “(O_2_CCH_2_Cl)]_10_, a Molecular Ferric Wheel,” Journal of the American Chemical Society 116 (1994): 823–832, 10.1021/ja00082a001.

[6] N. V. Gerbeleu , Y. T. Struchkov , G. A. Timko , A. S. Batsanov , K. M. Indrichan , and G. A. Popovich , “Octanuclear Chromium(III) Fluoride Trimethylacetate: A Complex Macrocycle,” Doklady Akademii Nauk SSSR 313 (1990): 1459.

[7] J. van Slageren , R. Sessoli , D. Gatteschi , et al., “Magnetic Anisotropy of the Antiferromagnetic Ring [Cr_8_F_8_Piv_16_],” Chemistry – A European Journal 8 (2002): 277. 10.1002/1521-3765(20020104)8:1<277::aid-chem277>3.0.co;2-d 11826863

[8] F. Meier and D. Loss , “Thermodynamics and Spin‐Tunneling Dynamics in Ferric Wheels With Excess Spin,” Physical Review B 64 (2001): 224411, 10.1103/PhysRevB.64.224411.

[9] F. K. Larsen , E. J. L. McInnes , H. El Mkami , et al., “Synthesis and Characterization of Heterometallic {Cr_7_M} Wheels,” Angewandte Chemie International Edition 42 (2003): 101. 10.1002/anie.200390034 19757603

[10] E. J. L. McInnes , G. A. Timco , G. F. S. Whitehead , and R. E. P. Winpenny , “Heterometallic Rings: Their Physics and Use as Supramolecular Building Blocks,” Angewandte Chemie International Edition 54 (2015): 14244–14269, 10.1002/anie.201502730. 26459810

[11] F. Troiani , A. Ghirri , M. Affronte , et al., “Molecular Engineering of Antiferromagnetic Rings for Quantum Computation,” Physical Review Letters 94 (2005): 207208, 10.1103/PhysRevLett.94.207208. 16090284

[12] A. Ardavan , O. Rival , J. J. L. Morton , et al., “Will Spin‐Relaxation Times in Molecular Magnets Permit Quantum Information Processing?,” Physical Review Letters 98 (2007): 057201, 10.1103/PhysRevLett.98.057201. 17358891

[13] K. Bader , D. Dengler , S. Lenz , et al., “Room Temperature Quantum Coherence in a Potential Molecular Qubit,” Nature Communications 5 (2014): 5304, 10.1038/ncomms6304. 25328006

[14] M. J. Graham , J. M. Zadrozny , M. Shiddiq , et al., “Influence of Electronic Spin and Spin–Orbit Coupling on Decoherence in Mononuclear Transition Metal Complexes,” Journal of the American Chemical Society 136 (2014): 7623–7626, 10.1021/ja5037397. 24836983

[15] S. H. Hansen , C. D. Buch , J. B. Petersen , et al., “Probing Decoherence in Molecular 4f Qubits,” Chemical Science 15 (2024): 20329. 10.1039/d4sc05304d PMC11575486 39568949

[16] M. Atzori , L. Tesi , E. Morra , M. Chiesa , L. Sorace , and R. Sessoli , “Room‐Temperature Quantum Coherence and Rabi Oscillations in Vanadyl Phthalocyanine: toward Multifunctional Molecular Spin Qubits,” Journal of the American Chemical Society 138 (2016): 2154–2157, 10.1021/jacs.5b13408. 26853512

[17] A.‐M. Ariciu , D. H. Woen , D. N. Huh , et al., “Engineering Electronic Structure to Prolong Relaxation Times in Molecular Qubits by Minimising Orbital Angular Momentum,” Nature Communications 10 (2019): 3330, 10.1038/s41467-019-11309-3. PMC6659626 31350411

[18] S. Wisbeck , A. L. Sorrentino , F. S. Santana , et al., “(η^8^‐Cyclooctatetraene)(η^5^‐fluorenyl)titanium: A Processable Molecular Spin Qubit With Optimized Control of the Molecule–Substrate Interface,” Chemical Science 15 (2024): 14390. 10.1039/d4sc03290j PMC11325857 39156928

[19] A. Ardavan , A. Bowen , A. Fernandez , et al., “Engineering Coherent Interactions in Molecular Nanomagnet Dimers,” NPJ Quantum Information 1 (2015): 15012, 10.1038/npjqi.2015.12.

[20] J. Ferrando‐Soria , E. M. Pineda , A. Chiesa , et al., “A Modular Design of Molecular Qubits to Implement Universal Quantum Gates,” Nature Communications 7 (2016): 11377, 10.1038/ncomms11377. PMC4848482 27109358

[21] S. J. Lockyer , A. Chiesa , A. Brookfield , et al., “Five‐Spin Supramolecule for Simulating Quantum Decoherence of Bell States,” Journal of the American Chemical Society 144 (2022): 16086–16092, 10.1021/jacs.2c06384. PMC9460766 36007954

[22] J. Ferrando‐Soria , S. A. Magee , A. Chiesa , et al., “Switchable Interaction in Molecular Double Qubits,” Chemistry 1 (2016): 727.

[23] S. J. Lockyer , L. Loci , A. Brookfield , et al., “Extending the Distance Range in Double Electron–Electron Resonance Measurements of Transition Metal Clusters,” Journal of the American Chemical Society 148 (2026): 2896. 10.1021/jacs.5c20715 41527890

[24] S. Nakazawa , S. Nishida , T. Ise , et al., “A Synthetic Two‐Spin Quantum Bit: *G*‐Engineered Exchange‐Coupled Biradical Designed for Controlled‐NOT Gate Operations,” Angewandte Chemie International Edition 51 (2012): 9860. 10.1002/anie.201204489 22936609

[25] D. Aguila , L. A. Barrios , V. Velasco , et al., “Heterodimetallic [LnLn′] Lanthanide Complexes: Toward a Chemical Design of Two‐Qubit Molecular Spin Quantum Gates,” Journal of the American Chemical Society 136 (2014): 14215–14222, 10.1021/ja507809w. PMC4195387 25203521

[26] G. Aromí , D. Aquila , P. Gamez , F. Luis , and O. Roubeau , “Design of Magnetic Coordination Complexes for Quantum Computing,” Chemical Society Reviews 41 (2012): 537. 10.1039/c1cs15115k 21818467

[27] H. Mao , G. J. Pažera , M. Krzyaniak , and M. R. Wasielewski , “Quantum Gate Operations on a Spectrally Addressable Photogenerated Molecular Electron Spin‐Qubit Pair,” Journal of the American Chemical Society 145 (2023): 6585–6593, 10.1021/jacs.3c01243. 36913602

[28] A. Privitera , A. Chiesa , F. Santanni , et al., “Room‐temperature Optical Spin Polarization of an Electron Spin Qudit in a Vanadyl‐Free Base Porphyrin Dimer,” Journal of the American Chemical Society 147 (2025): 331–341, 10.1021/jacs.4c10632. PMC11726572 39681297

[29] M. Dan‐Hardi , C. Serre , T. Frot , et al., “A New Photoactive Crystalline Highly Porous Titanium(IV) Dicarboxylate,” Journal of the American Chemical Society 131 (2009): 10857–10859, 10.1021/ja903726m. 19621926

[30] G. A. Timco , A. Fernandez , A. K. Kostopoulos , et al., “An Extensive Family of Heterometallic Titanium(IV)–Metal(III) Rings With Structure Control Through Templates,” Angewandte Chemie International Edition 56 (2017): 13629. 10.1002/anie.201706679 28884939

[31] H. Barrow , D. A. Brown , N. W. Alcock , H. J. Clase , and M. G. H. Wallbridge , “An Octameric Titanium Oxo Metallacycle With Host–Guest Interactions,” Journal of the Chemical Society, Chemical Communications 12 (1995): 1231.

[32] H. Kusama and H. Arakawa , “Influence of the Porous Structure of TiO_2_ Electrodes on the Performance of Dye‐Sensitized Solar Cells,” Solar Energy Materials Solar Cells 81 (2004): 87.

[33] Deposition numbers 2502491 (**3**) and 2502492 (4) contain the supplementary crystallographic data for this paper. These data are provided free of charge by the joint Cambridge Crystallographic Data Centre and Fachinformationszentrum Karlsruhe Access Structures service.

[34] S. J. Lockyer , A. Chiesa , G. A. Timco , et al., “Targeting Molecular Quantum Memory With Embedded Error Correction,” Chemical Science 12 (2021): 9104. 10.1039/d1sc01506k PMC8261727 34276940

[35] E. Garlatti , M. A. Albring , M. L. Baker , R. J. Docherty , H. Mutka , T. Guidi , V. Garcia‐Sakai , G. F. S. Whitehead , R. G. Pritchard , G. A. Timco , F. Tuna , G. Amoretti , S. Carretta , P. Santini , G. Lorusso , M. Affronte , E. J. L. McInnes , D. Collison , and R. E. P. Winpenny , “A Detailed Study of the Magnetism of Chiral {Cr_7_M} Rings: an Investigation Into Parametrization and Transferability of Parameters,” Journal of the American Chemical Society 136 (2014): 9763. 10.1021/ja5047445 24968057

[36] K. D. Demadis , C. M. Hartson , and T. J. Meyer , “The Localized‐to‐Delocalized Transition in Mixed‐Valence Chemistry,” Chemical Reviews 101 (2001): 2655–2686, 10.1021/cr990413m. 11749392

[37] D. M. D'Alessandro and F. R. Keene , “Intervalence Charge Transfer (IVCT) in Trinuclear and Tetranuclear Complexes of Iron, Ruthenium, and Osmium,” Chemical Reviews 106 (2006): 2270. 10.1021/cr050010o 16771450

[38] M. B. Robin and P. Day , “Mixed Valence Chemistry‐A Survey and Classification,” Advances in Inorganic Chemistry and Radiochemistry 10 (1967): 247.

[39] M. A. Nasalevich , C. H. Hendon , J. G. Santaclara , K. Svane , B. van der Linden , S. L. Veber , M. V. Fedin , A. J. Houtepen , M. A. can der Veen , F. Kapteijn , A. Walsh , and J. Gascon , “Electronic Origins of Photocatalytic Activity in D^0^ Metal Organic Frameworks,” Scientific Reports 6 (2016): 23676. 10.1038/srep23676 PMC4810359 27020767

[40] S. E. Brown , I. Mantaloufa , R. T. Andrews , T. J. Barnes , M. R. Lees , F. De Proft , A. V. Cunha , and S. D. Pike , “Photoactivation of Titanium‐oxo Cluster [Ti_6_O_6_(OR)_6_(O_2_CtBu)_6_]: Mechanism, Photoactivated Structures, and Onward Reactivity With O_2_ to a Peroxide Complex, Photoactivated Structures, and Onward Reactivity With O_2_ to a Peroxide Complex,” Chemical Science 14 (2023): 675–683, 10.1039/D2SC05671B. PMC9847671 36741534

[41] S. Piligkos , H. Weihe , E. Bill , F. Neese , H. El Mkami , G. M. Smith , D. Collison , G. Rajaraman , G. A. Timco , R. E. P. Winpenny , and E. J. L. McInnes , “EPR Spectroscopy of a Family of Cr^III^ _7_M^II^ (M = Cd, Zn, Mn, Ni) "Wheels": Studies of Isostructural Compounds With Different Spin Ground States,” Chemistry – A European Journal 15 (2009): 3152. 10.1002/chem.200801895 19199301

[42] M. Chiesa , M. C. Paganini , S. Livraghi , and E. Giamello , “Charge Trapping in TiO_2_ Polymorphs as Seen by Electron Paramagnetic Resonance Spectroscopy,” Physical Chemistry Chemical Physics 15 (2013): 9435. 10.1039/c3cp50658d 23695705

[43] J. J. Davies and J. E. Wertz , “The EPR Spectrum of Trivalent Titanium in Orthorhombic Symmetry in MgO,” Journal of Physics and Chemistry of Solids 31 (1970): 2489–2494, 10.1016/0022-3697(70)90066-1.

[44] S. S. Eaton and G. R. Eaton , “Relaxation Times of Organic Radicals and Transition Metal Ions,” Biological Magnetic Resonance 19 (2002): 29.

[45] S. M. Jahn , R. K. Stowell , and S. Stoll , “The Contribution of Methyl Groups to Electron Spin Decoherence of Nitroxides in Glassy Matrices,” Journal of Chemical Physics 161 (2024): 174119, 10.1063/5.0240801. PMC11556899 39503471

[46] C. J. Wedge , R. E. George , G. A. Timco , F. Tuna , S. Rigby , E. J. L. McInnes , R. E. P. Winpenny , S. J. Blundell , and A. Ardavan , “Chemical Engineering of Molecular Qubits,” Physical Review Letters 108 (2012): 107204, 10.1103/PhysRevLett.108.107204. 22463450

[47] G. M. Sheldrick , “SADABS, Empirical Absorption Correction Program Based Upon the Method of Blessing,” Journal of Applied Crystallography 48 (2015): 1907.

[48] L. Krause , R. Herbst‐Irmer , G. M. Sheldrick , and D. Stalke , “An Empirical Correction for the Influence of Low‐Energy Contamination,” Journal of Applied Crystallography 48 (2015): 1907–1913, 10.1107/S1600576715020440. PMC4453166 26089746

[49] R. H. Blessing , “An Empirical Correction for Absorption Anisotropy,” Acta Crystallographica Section A 51 (1995): 33. 10.1107/s0108767394005726 7702794

[50] G. M. Sheldrick , “Crystal Structure Refinement With SHELXL,” Acta Crystallographica Section C 71 (2015): 3. 10.1107/S2053229614024218 PMC4294323 25567568

[51] O. V. Dolomanov , L. J. Bourhis , R. J. Gildea , J. A. K. Howard , and H. Puschmann , “OLEX2: A Complete Structure Solution, Refinement and Analysis Program,” Journal of Applied Crystallography 42 (2009): 339–341, 10.1107/S0021889808042726.

[52] S. Stoll and A. Schweiger , “EasySpin, a Comprehensive Software Package for Spectral Simulation and Analysis in EPR,” Journal of Magnetic Resonance 178 (2006): 42–55, 10.1016/j.jmr.2005.08.013. 16188474

