# Supplementary material for: Heterovalent, Homometallic Rings: Synthesis, Optical, and EPR Studies of {TiIV 7TiIII} Complexes
Source: Chemistry. 2026 Jul 18;32(39):e71451. doi: 10.1002/chem.71451 (PMC13715050; doi:10.1002/chem.71451)
Supplement: Supplementary file 1 [file CHEM-32-e71451-s001.pdf]

Supporting Information

Homometallic, heterovalent rings: synthesis, optical and EPR studies of $\{Ti^{IV}{}_7Ti^{III}\}$ complexes.

Selena J. Lockyer,* Lubomir Loci, Yingzhao Ma, Matthew D. Stevens, Grigore A. Timco, George F. S. Whitehead, Richard E. P. Winpenny, Alice M. Bowen and Eric J. L. McInnes*

School of Chemistry and Photon Science Institute, The University of Manchester, Oxford Road, Manchester M13 9PL, U.K.

CONTENTS:

1. Experimental Section S2

1.1 Synthesis of **1** and **2**. S2

1.2 Synthesis of **3**. S2

1.3 Synthesis of **4**. S3

2. Crystallography S3

3. Structures S5

4. UV-Vis / NIR S6

5. Electron Paramagnetic Resonance Measurements S7

5.1 Continuous Wave EPR. S7

5.2 Pulse EPR. . S15

5.2.1 Echo Detected Field Sweeps. S15

5.2.2 Phase Memory Times (T_m). S16

5.2.3 Spin Lattice Relaxation Times (T_1). S26

5.2.4 Transient Nutation S33

6. References S37

1. Experimental Section

General remarks: All starting reagents and materials used were sourced from TCI, Sigma-Aldrich and/or Fluorochem. Unless stated otherwise, all reagents and solvents were used without further purification. Where stated the compounds were synthesized and crystallised under an inert atmosphere in anhydrous solvents, using standard Schlenk line techniques. Chemical shifts are reported in parts per million (ppm) from low to high frequency and referenced to the residual solvent resonance. ESI mass spectrometry and microanalysis were carried out by the services at The University of Manchester.

Synthetic methods

1.1 Compounds **1** $(PyCH_2NH_2CH_2CH_3)[Cr_7NiF_8(O_2C^tBu)_{16}]\}$ and **2** $(PyCH_2NH_2CH_2CH_3)[Cr_7NiF_8(O_2CHC_2H_4)_{16}]$ were prepared as reported previously.[23]

1.2 Synthesis of **3, $(PyCH_2NH_2CH_2CH_3)[Ti_7TiO_8(O_2C^tBu)_{16}]$:**

Pivalic acid (18 g, 176 mmol) and 4-(ethylaminomethyl)pyridine (1g, 7.2 mmol) were placed under an inert atmosphere and heated to 60 °C until all dissolved. Titanium(IV) isopropoxide (2.5 mL, 8.5 mmol) was added and the mixture heated to 140 °C for 20 hours. The solution was then cooled to room temperature and acetonitrile (100 mL) added to precipitate the product. The supernatant was then filtered off and the remaining solid washed twice with acetonitrile (2 x 50 mL). The solid was dried under vacuum, redissolved in toluene (20 mL), concentrated to ca. 5 mL, then left at 5 °C to produce blue crystals over 2-3 days. Yield: 1.10 g (46%).

Elemental analysis %: calc. for $C_{88}H_{157}N_2O_{40}Ti$: C 46.64, H 6.98, N 1.24; found: C 46.64, H 7.18 N 1.35.

1.3 Synthesis of **4, ($(PyCH_2NH_2CH_2CH_3)[Ti_7TiO_8(O_2CC_3H_5)_{16}]$):**

Cyclopropanecarboxylic acid (23 mL, 294 mmol), and 4-(ethylaminomethyl)pyridine (1g, 7.2 mmol) were placed under an inert atmosphere and heated to 60 °C until all dissolved. Titanium(IV) isopropoxide (7.0 mL, 17.5 mmol) was added and the mixture heated to 140 °C for 24 hours. Maintaining a temperature of 140°C, a high vacuum (ca. 10^{-2} mbar) was applied, and all volatiles removed *in vacuo*. The residue was then dissolved in dichloromethane (10 mL), then (100 mL) of hexane was added with stirring to precipitate the product. The supernatant was filtered off, and the solids dried under vacuum. The powder was dissolved in dichloromethane (5 mL) and liquid diffusion with hexane (5 mL) was used to obtain small dark blue crystals. Yield: 1.28 g (30%). ESI MS m/z (relative intensity) 2009 $[M+Na]^+$. Elemental analysis %: calc. for $C_{72}H_{93}N_2O_{40}Ti_8$: C 43.04, H 4.66, N 1.39; found: C 42.90, H 4.78 N 1.33.

2. <u>Crystallography</u>

Data Collection:

X-ray diffraction data for compounds **3** and **4**, were collected at a temperature of 100 K and 150 K, respectively. Using a Rigaku FR-X with Cu-Kα radiation equipped with a Hypix 6000HE detector, equipped with an Oxford Cryosystems nitrogen flow gas system. Data was measured using CrysAlisPro suite of programs.

Crystal structure determinations and refinements:

X-Ray data were processed and reduced using the CrysAlisPro suite of programs. Absorption correction was performed using empirical methods (SCALE3 ABSPACK) based upon symmetry-equivalent reflections combined with measurements at different azimuthal angles.[S1] The crystal structure was solved and refined against all F^2 values using the SHELXL and Olex 2 suite of programmes.[S2]

CCDC 2502491 and 2502492 contain the supplementary crystallographic data for **3** and **4**, respectively. These data can be obtained free of charge via https://www.ccdc.cam.ac.uk/structures/, or by emailing data_request@ccdc.cam.ac.uk, or by contacting The Cambridge Crystallographic Data Centre, 12 Union Road, Cambridge, CB2 1EZ, UK; fax: +44 1223 336033.

Table S1. Crystal data: Compounds **3** and **4**

**Parameter**	**3**	**4**
Identification code	lrepw650	lrepw640
Empirical formula	$C_{119.5}H_{193}N_2O_{40}Ti_8$	$C_{73}H_{95}Cl_2N_2O_{40}Ti_8$
Formula weight	2680.95	2094.60
Temperature/K	99.9(3)	150.00(10)
Crystal system	triclinic	monoclinic
Space group	P-1	$P2_1$
a/Å	22.437(2)	18.4405(3)
b/Å	22.965(2)	13.43242(18)
c/Å	29.6047(11)	18.8566(3)
α/°	90.983(5)	90
β/°	91.297(5)	98.0684(16)
γ/°	97.862(8)	90
Volume/Å^3	15104(2)	4624.57(12)
Z	4	2
ρ_{calc}g/cm^3	1.179	1.504
μ/mm^{-1}	3.988	6.868
F(000)	5680.0	2154.0
Crystal size/mm^3	0.422 × 0.41 × 0.095	0.38 × 0.31 × 0.215
Radiation	Cu Kα (λ = 1.54184)	Cu Kα (λ = 1.54184)
2Θ range for data collection/°	2.986 to 71.464	4.734 to 136.496
Index ranges	-16 ≤ h ≤ 16, -17 ≤ k ≤ 17, -22 ≤ l ≤ 22	-21 ≤ h ≤ 22, -16 ≤ k ≤ 16, -22 ≤ l ≤ 22
Reflections collected	45224	52804
Independent reflections	13717 [R_{int} = 0.0875, R_{sigma} = 0.0647]	16500 [R_{int} = 0.0464, R_{sigma} = 0.0529]
Data/restraints/parameters	13717/8313/1585	16500/2839/1365
Goodness-of-fit on F^2	2.020	1.034
Final R indexes [I>=2σ (I)]	R_1 = 0.1805, wR_2 = 0.4648	R_1 = 0.0694, wR_2 = 0.1946
Final R indexes [all data]	R_1 = 0.2085, wR_2 = 0.4864	R_1 = 0.0738, wR_2 = 0.2034
Largest diff. peak/hole / e Å^{-3}	0.79/-0.61	0.62/-0.59

3. Structures

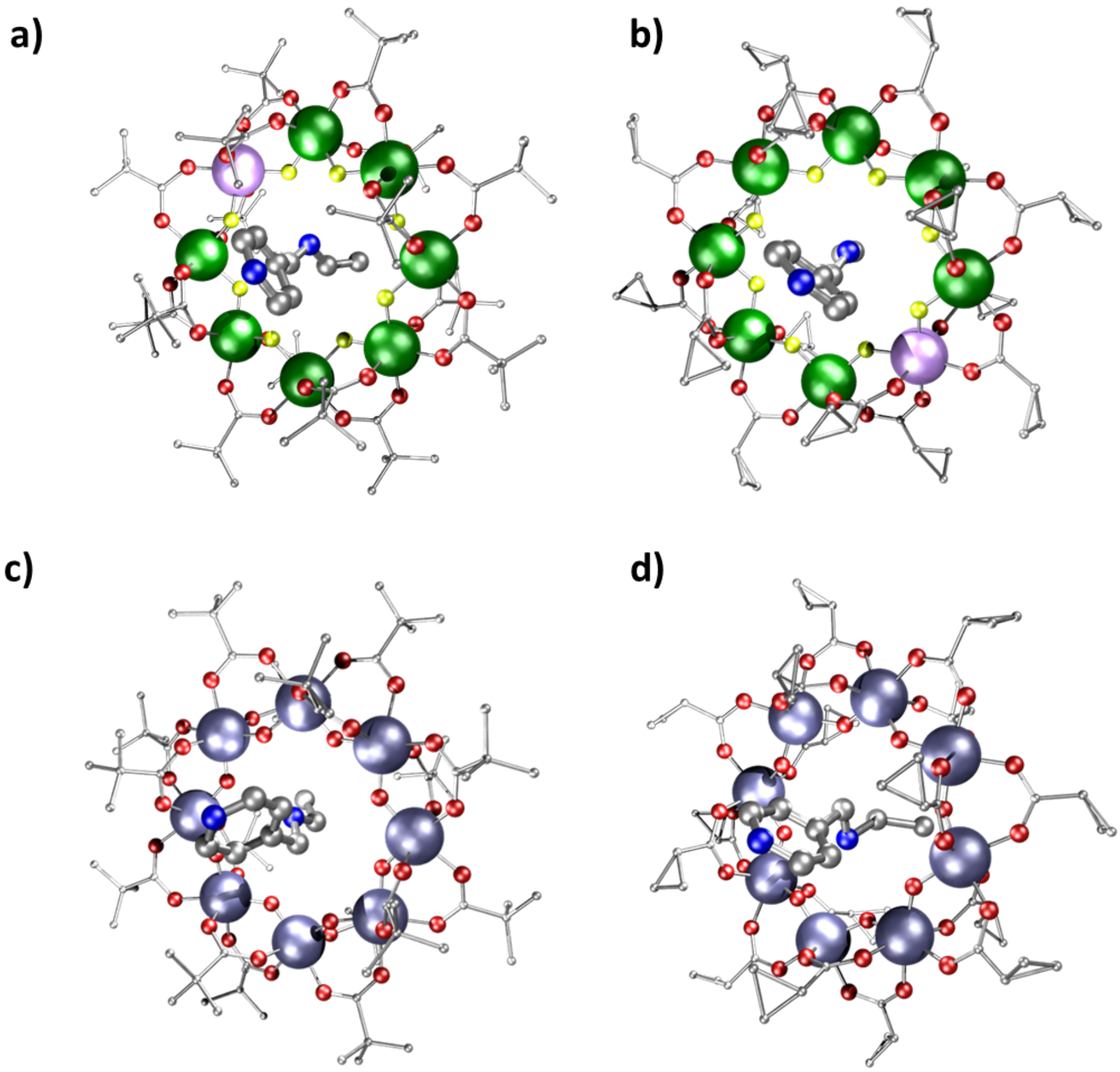

Figure S1. Crystal structures of a) **1**, b) **2**, c) **3** and d) **4**. Atom colours: blue (N), red (O), grey (C), green (Cr), purple (Ni), dark blue (Ti). Hydrogens omitted for clarity.

Table S2. Metal bond distances and angles for compounds **1 – 4.**

Compound	Metal-Oxo/Fluro Distance (Å)	Metal-Oxo/Fluro-Metal Angle (°)
1	1.93(0.1)	123.28(0.5)
2	1.92(0.1)	125.06(0.5)
3	1.82(0.1)	132.91(0.5)
4	1.82(0.1)	133.60(0.5)

4. UV-Vis / NIR

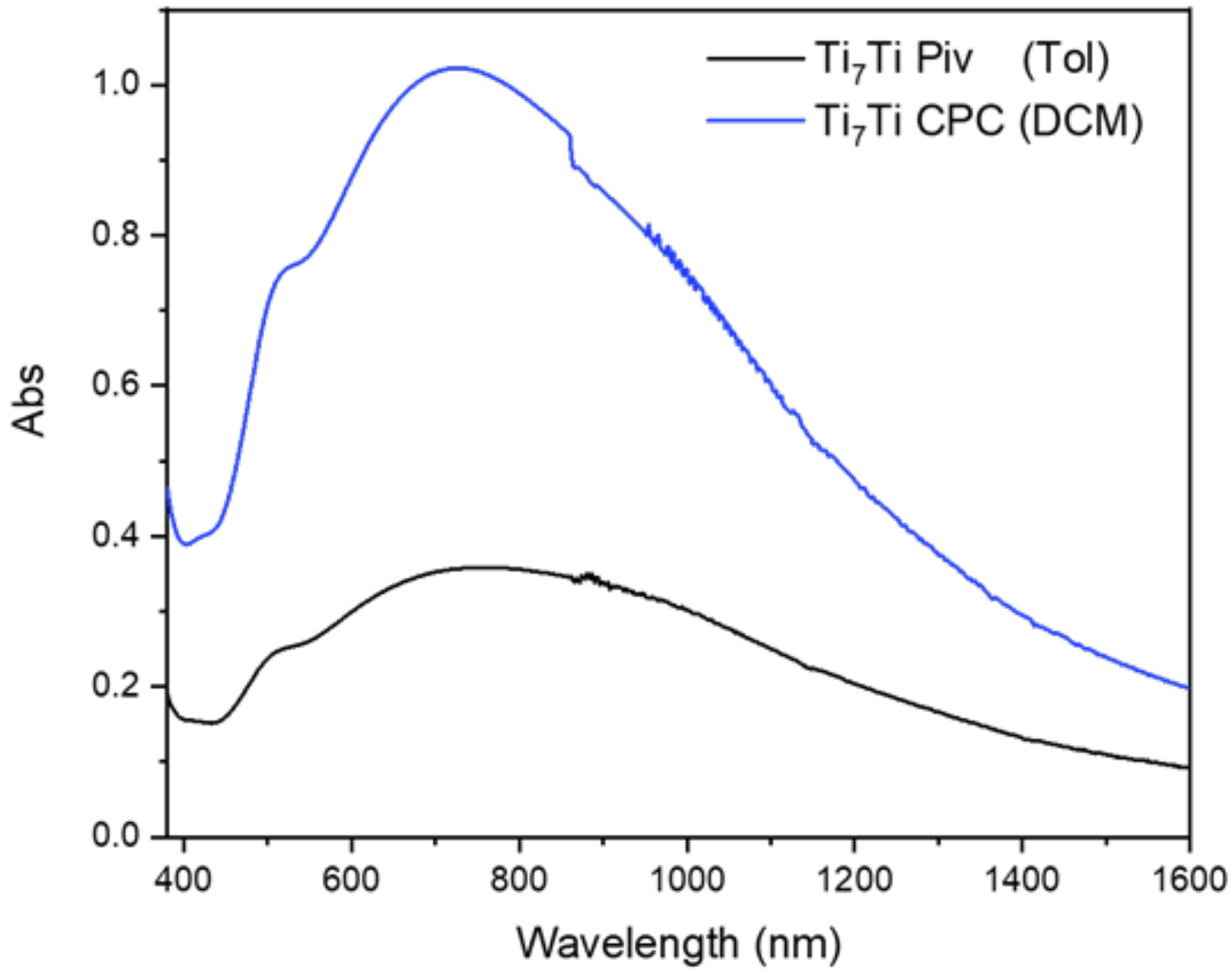

Figure S2. UV/Vis/NIR for **3** (black line) in toluene and **4** (blue line) in DCM. Spectra measured on 5 mM solutions, at room temperature, in a J Young's quartz cuvette with path length *l* = 3 mm. Assignment: λ_{max} at ca. 500 and 420 nm.

5. Electron Paramagnetic Resonance Measurements

5.1 Continuous Wave EPR:

Continuous wave (cw) X-band (~9.3 GHz) Q-band (~34 GHz) EPR spectra were recorded with a *Bruker EMXPlus* spectrometer equipped with a *Bruker ER5106QT flexline* resonator. Cryogenic temperatures were achieved using a *Bruker Stringer* closed cycle helium cryocooler mated to an *Oxford Instruments CF935 cryostat*. Temperature holding and regulation was controlled using an *Oxford Instruments MercuryITC*. The cw data were collected on polycrystalline powders and on 2 mM solutions (unless otherwise stated). Solvents were DCM and toluene (1:1) for **1** and **2**, toluene for **3** and DCM for **4**. All solvents were dried and degassed.

All cw spectra were field corrected using a *Bruker* 'Strong Pitch' standard (g = 2.0028) and all powder samples were checked for polycrystalline effects by measuring multiple random rotations. For display, cw X- and Q-band spectra were frequency corrected to 9.36 GHz and 34 GHz, respectively.

Spectral simulations were performed using the EasySpin 6.0.0 software[S3] with a spin Hamilton incorporating the g-matrices only:

$$\hat{H} = \mu_B \hat{S}^{Ring} \cdot \mathbf{g}^{Ring} \cdot \mathbf{B}$$

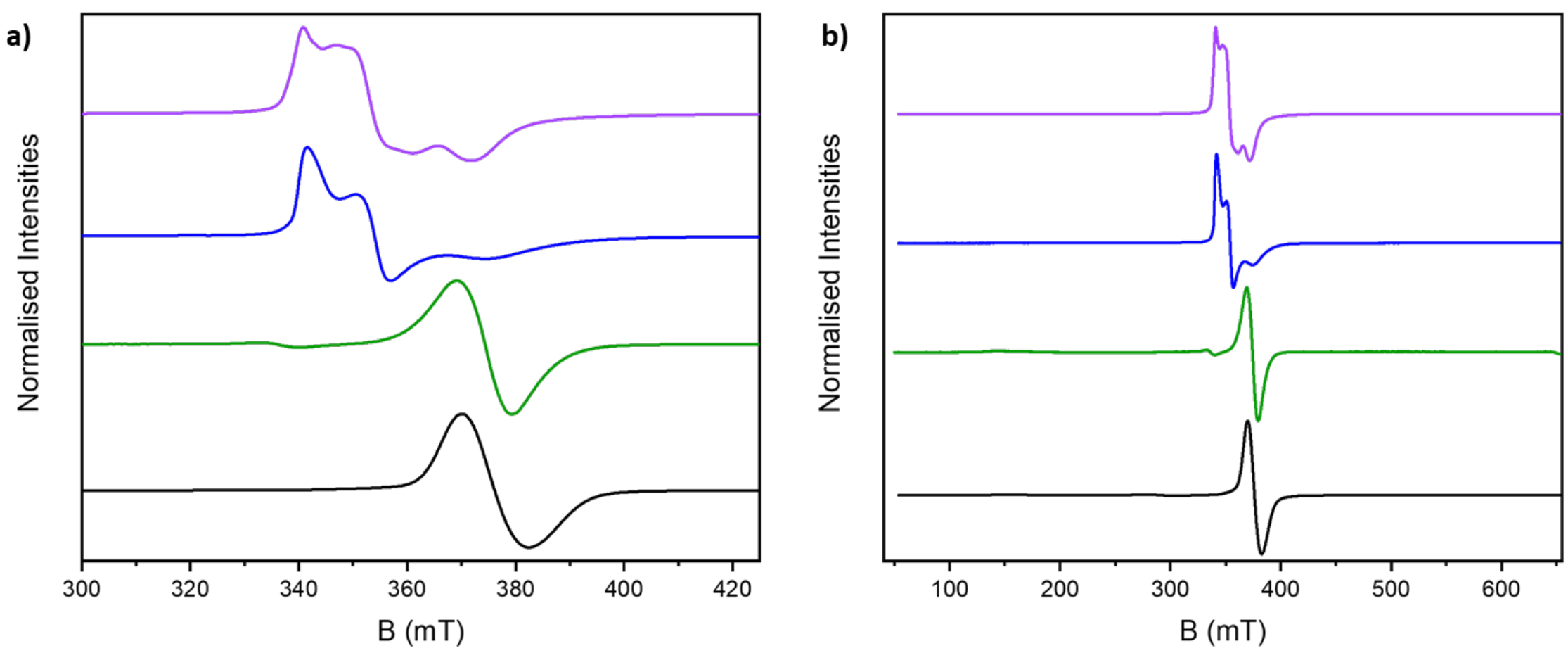

Figure S3. Cw X-band EPR spectra for **1** (black line), **2** (green), **3** (blue) and **4** (purple), performed on powder samples at 5 K: a) narrow field range 300 – 425 mT; b) wide field range 50 – 650 mT.

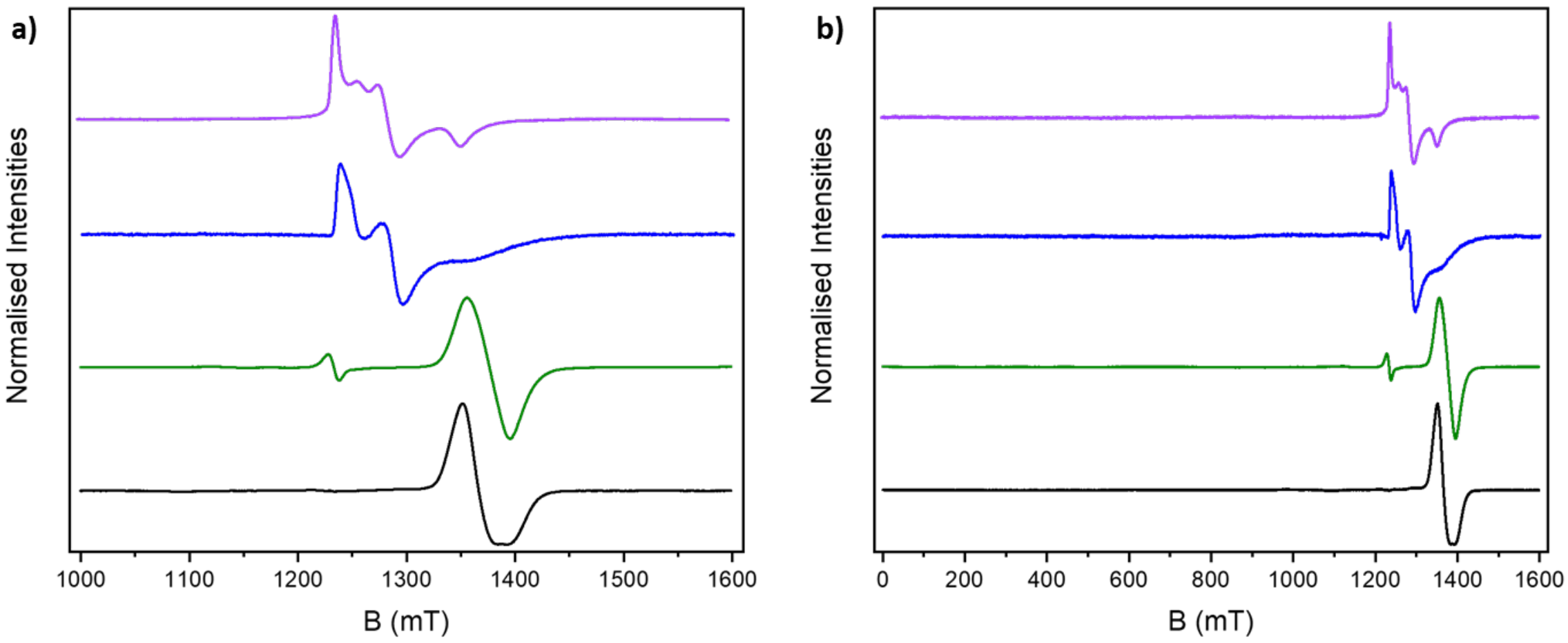

Figure S4. Cw Q-band EPR spectra for **1** (black line), **2** (green), **3** (blue) and **4** (purple) performed on powder samples at 5 K: a) narrow field range 1000 – 1600 mT; b) full field range 0 – 1600 mT.

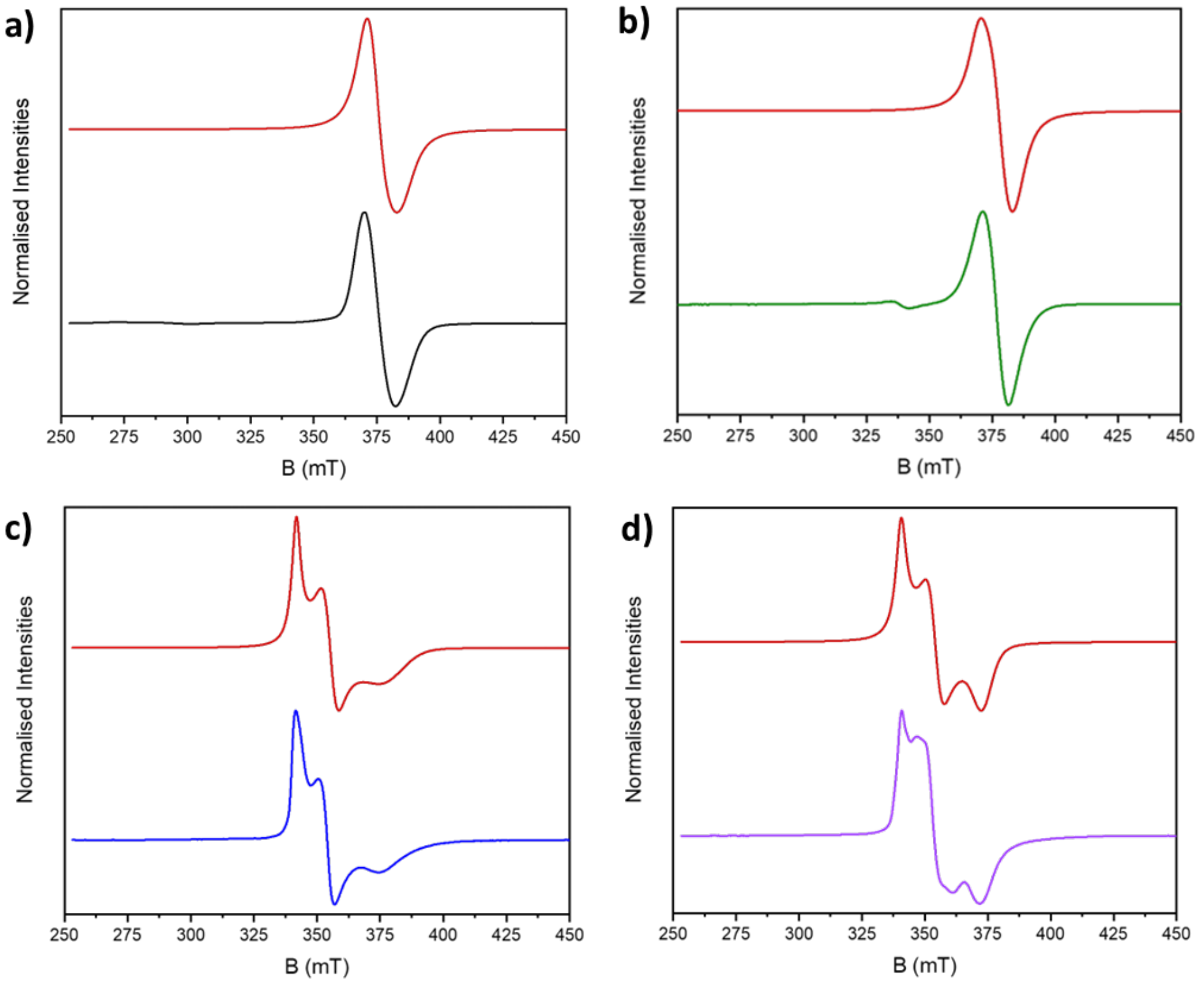

Figure S5. Cw X-band EPR spectra for power samples at 5 K for a) **1** (black), b) **2** (green), c) **3** (blue) and d) **4** (purple), with simulations (red lines). Simulation parameters are in Table S3.

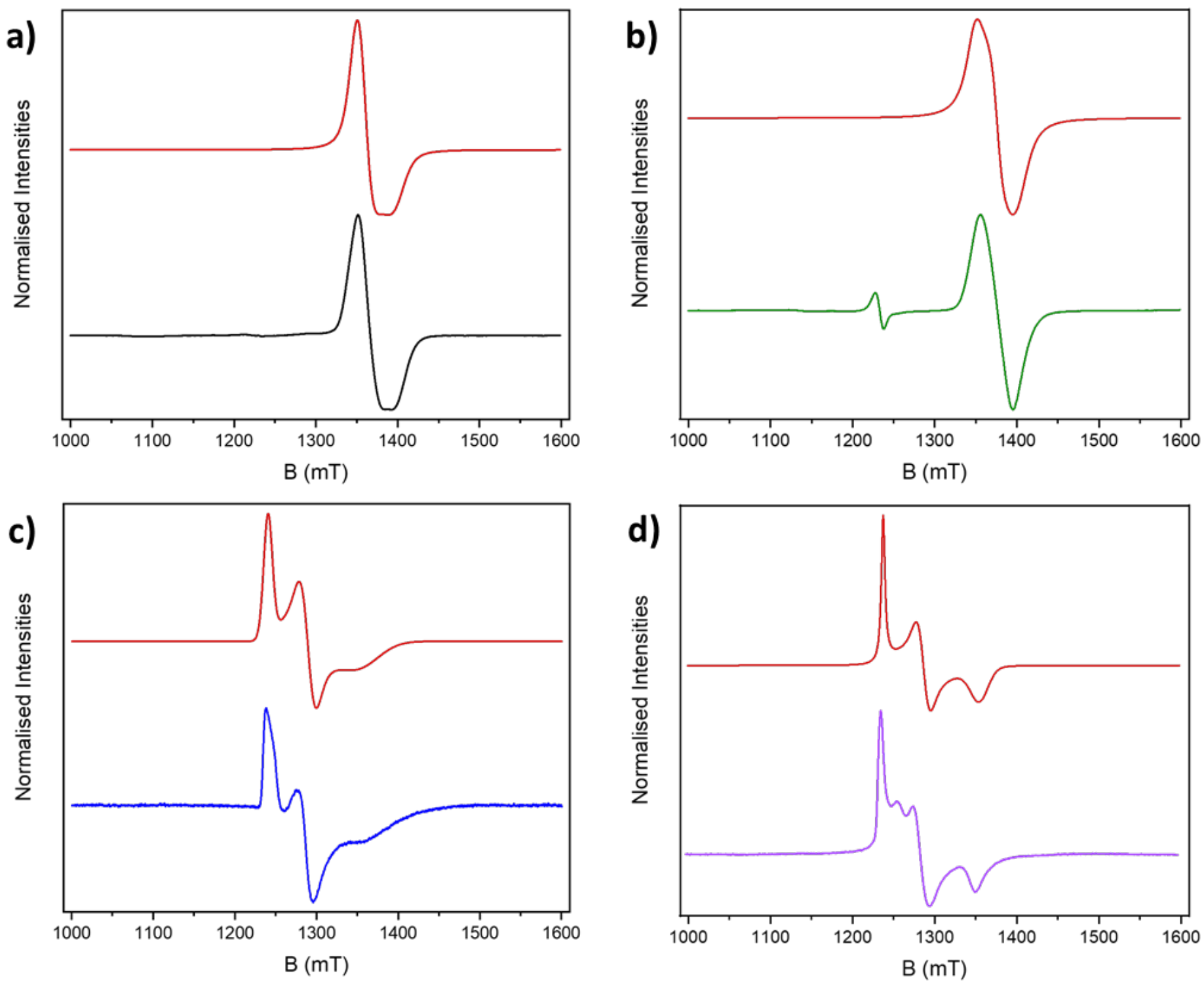

Figure S6. Cw Q-band EPR spectra for power samples at 5 K for a) **1** (black line), b) **2** (green), c) **3** (blue) and d) **4** (purple), with simulation (red line). Simulation parameters are in Table S3.

Table S3. Cw simulation parameters for **1 – 4**, as powder samples at 5 K (Figures S5 and S6).

Compound	g_{xyz}	g-Strain$_{xyz}$ / %	lwpp / mT (Lorentzian)
1	1.791, 1.791, 1.740,	2.2, 2.2, 2.8,	10
2	1.795, 1.795, 1.760,	2, 2, 2.5,	3
3	1.955, 1.880, 1.770,	0, 2, 7.5,	4
4	1.963, 1.888, 1.793,	0, 2, 3,	5

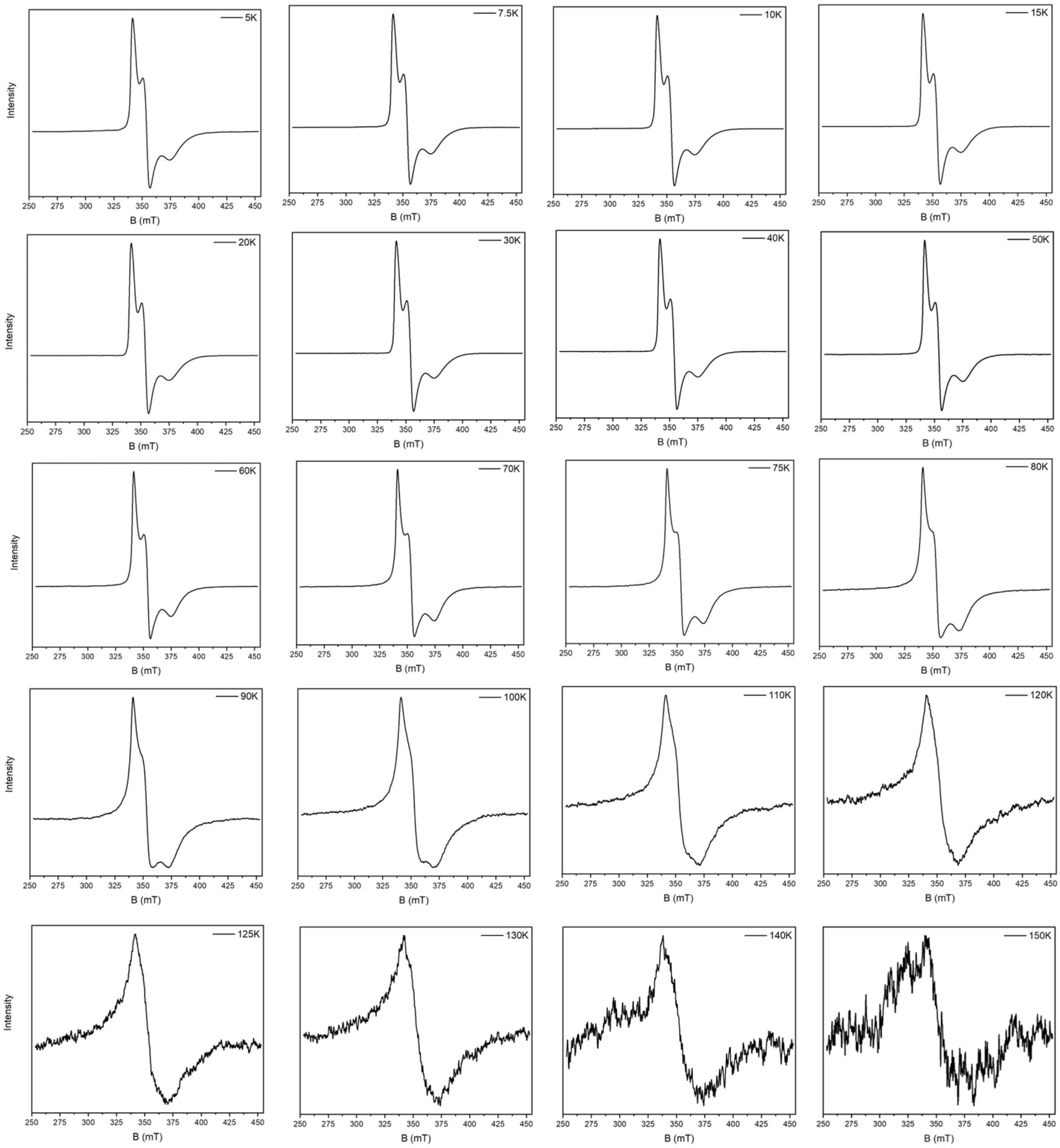

Figure S7. Cw Q-band variable temperature EPR spectra for power sample of **3**.

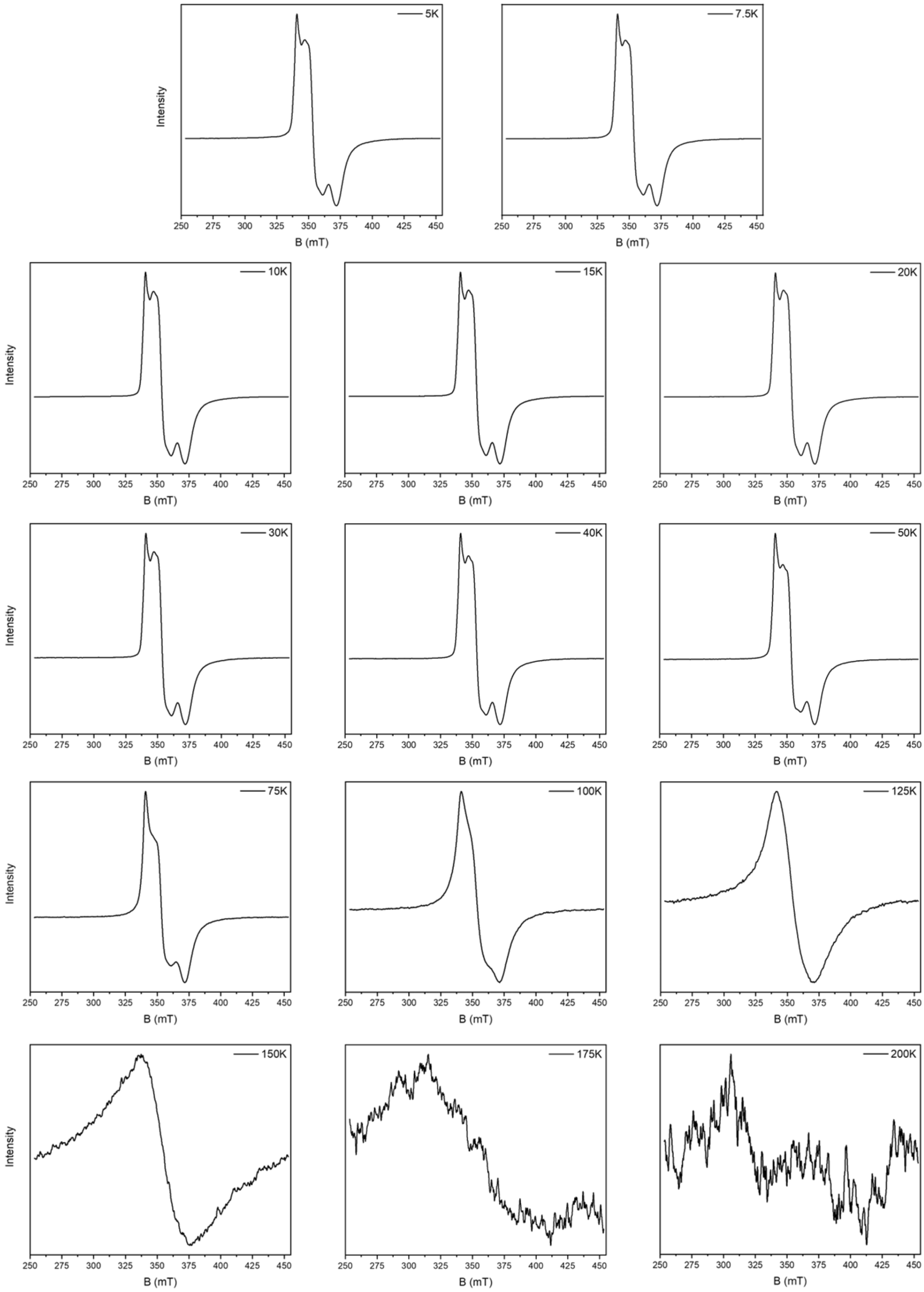

Figure S8. Cw X-band variable temperature EPR spectra for power sample of **4**.

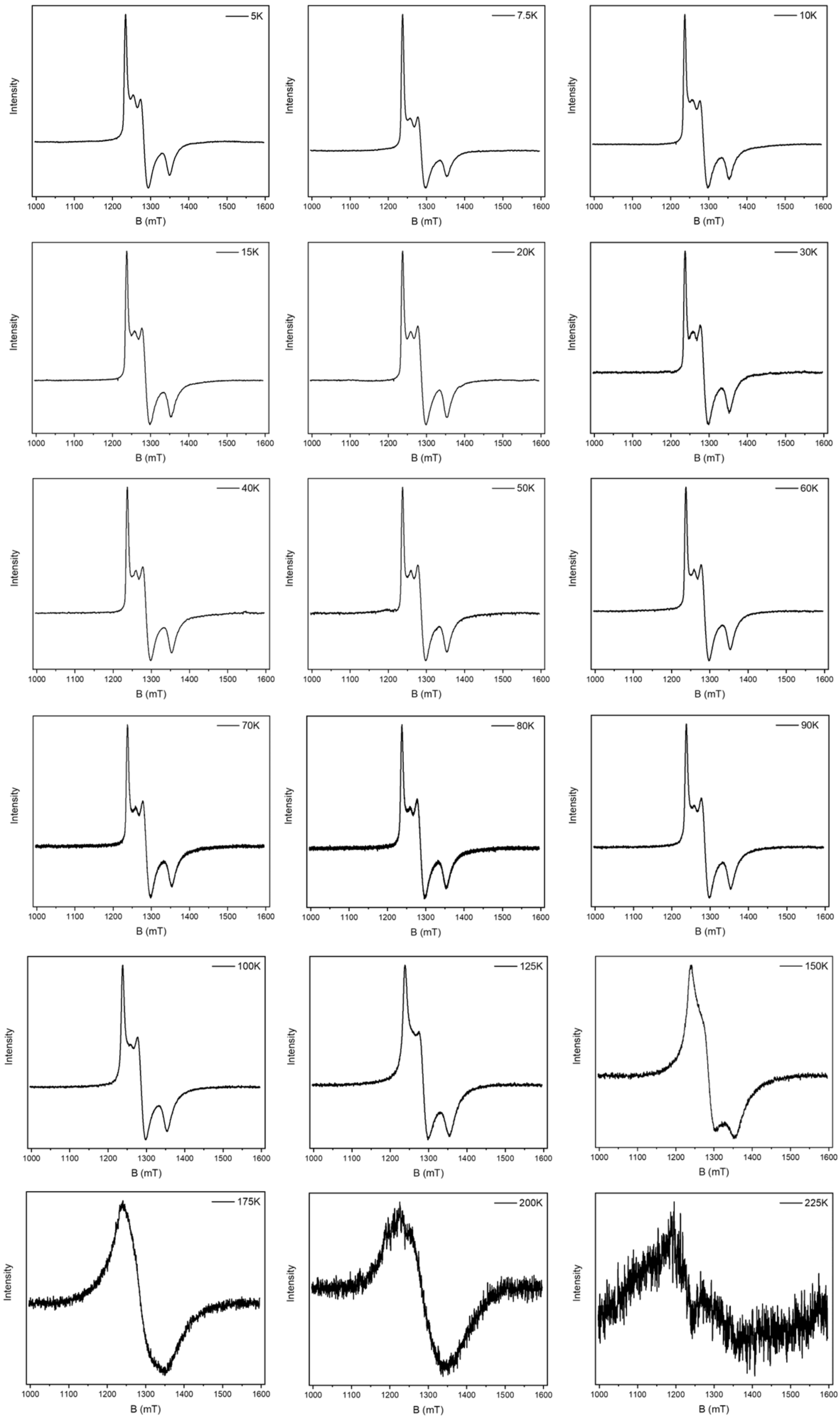

Figure S9. Cw Q-band variable temperature EPR spectra for power sample of **4**.

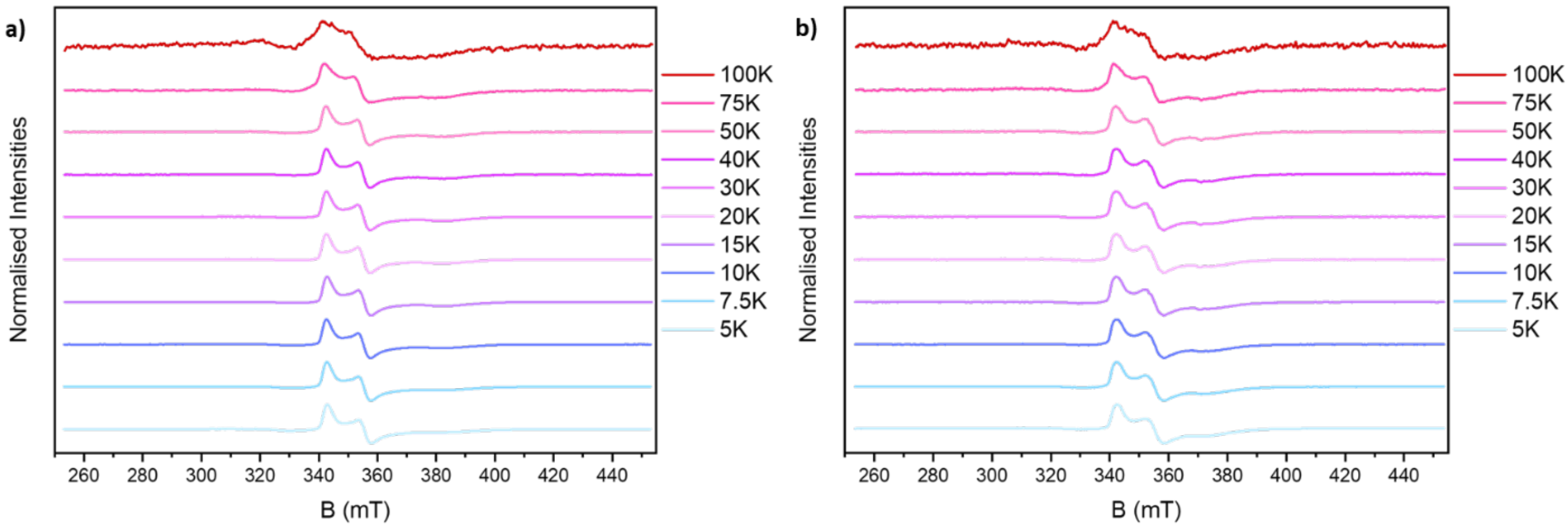

Figure S10. Cw X-band variable temperature EPR spectra for a) **3** and b) **4**, as 2 mM solutions.

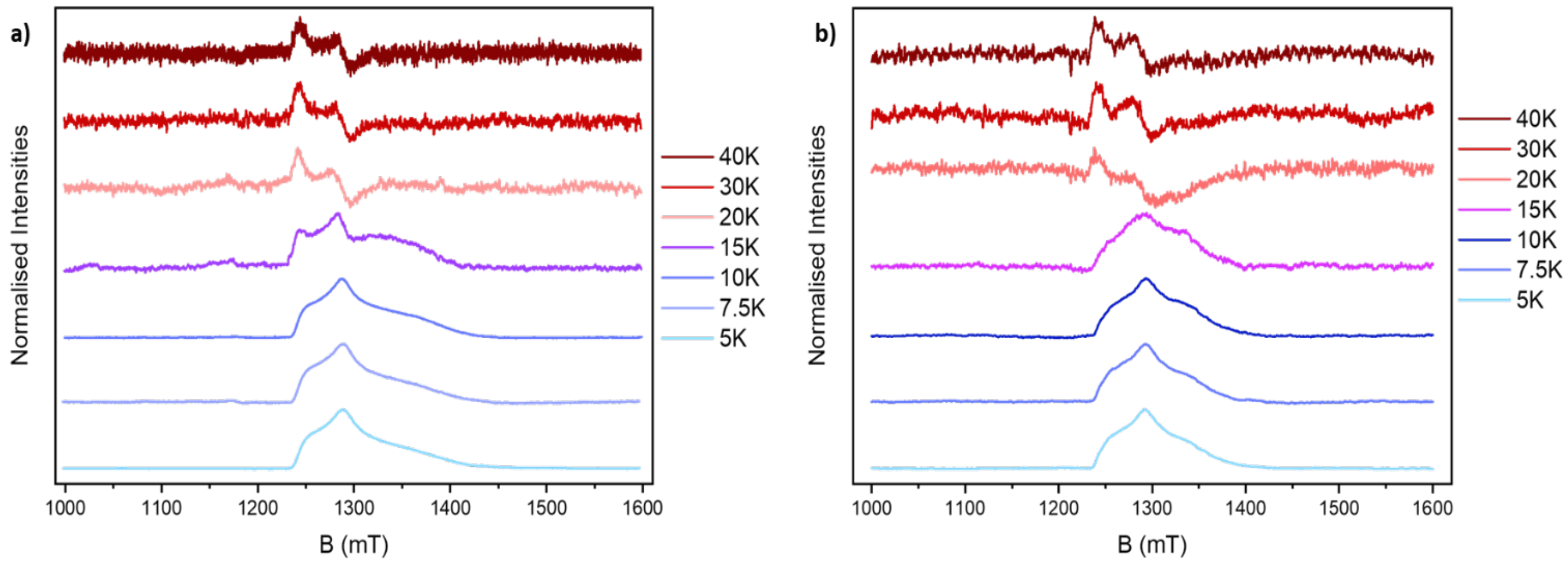

Figure S11. Cw Q-band variable temperature EPR spectra for a) **3** and b) **4** as 2 mM solutions.

5.2 Pulse EPR:

Pulsed X-band (9.7 GHz) EPR data were collected on a Bruker ELEXSYS E580X FT spectrometer. Pulsed Q-band (34 GHz) EPR data were collected on a Bruker ELEXSYS E580Q FT spectrometer. The data were collected from 0.2 mM solutions of dry and degassed toluene for **1**, **2**, **3** and DCM for **4**, at 3 K unless otherwise stated. All field positions were set to the signal maxima, unless otherwise stated.

5.2.1 Echo Detected Field Sweep (EDFS):

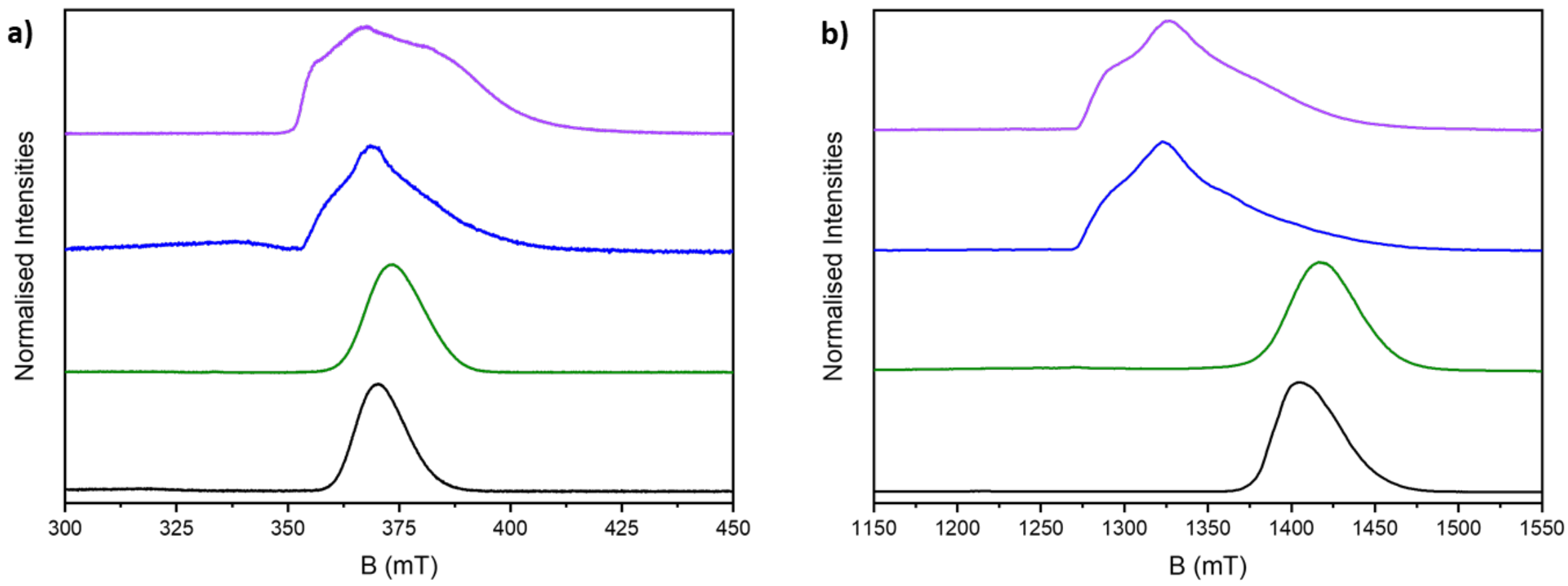

Figure S12. Echo Detected Field Sweep (EDFS) measurements performed for **1** (black line), **2** (green line), **3** (blue line) and **4** (purple line) at 3 K using a standard Hahn echo sequence π/2-*τ*-π-*τ*-echo. a) X-band and b) Q-band, using 20 and 40 ns pulses for π/2 and π, respectively and a *τ* = 300 ns.

5.2.2 Phase Memory Times (T_m):

Phase memory time constant (T_m) measurements were performed using a $\pi/2$-τ-π-τ-echo sequence. The echo decays were fit to an stretched (c) exponential decay with the form $I(2\tau) = I(2\tau_0)\exp[(-2\tau/T_m)^c]$. All pulse and τ lengths are given in the table below:

Table S4. Phase memory times measurement parameters:

Frequency	X-Band (9.7 GHz)					Q-Band (34 GHz)				
Compound	1	2	3		4	1	2	3		4
Temperature (K)	3-5	3-5	3-17	18-20	3-20	3-5	3-5	3-15	16-20	3-20
π/2 pulse (ns)	60	60	60	60	60	60	60	60	60	60
π pulse (ns)	120	120	120	120	120	120	120	120	120	120
τ (ns)	300	300	800	500	1000	300	300	1000	800	1000

Table S5. Phase memory time constants (T_m) for **1** and **2**.[23] Uncertainty from statistical fitting in parentheses; errors estimated from repeat measurements on different sample given as range. All measurements were performed at the EDFS maximum.

Compound	1		2	
	T_m / ns		T_m / ns	
Temperature / K	X-Band	Q-Band	X-Band	Q-Band
3	590(1)±12	533(0.5)	2146(6)±100	1908(1)
4	526(2)±35	479(0.5)	1650(6)±49	1290(1)
5	430(2)±38	400(0.5)	734(2)±170	724(4)

Table S6. Phase memory time constants (T_m) and stretch parameters (c) for **3** from Hahn echo decays. Uncertainty from statistical fitting in parentheses; errors estimated from repeat measurements on different sample given as range. All measurements were performed at the EDFS maximum.

Frequency	X-Band		Q-Band	
**Temperature / K**	**T_m / ns**	c	**T_m / ns**	c
**3**	1096(5)±44	1.161(3)±0.152	944(1)	0.912(8)
**4**	1080(4)±37	1.136(7)±0.128	932(1)	0.908(9)
**5**	1093(3)±39	1.156(5)±0.134	930(1)	0.910(1)
**6**	1052(2)±83	1.186(4)±0.167	919(1)	0.906(9)
**7**	1059(2)±51	1.179(4)±0.590	915(2)	0.907(1)
**8**	1049(2)±54	1.153(3)±0.144	906(2)	0.906(2)
**9**	1037(2)±53	1.152(4)±0.143	896(2)	0.907(1)
**10**	1017(2)±34	1.145(4)±0.164	884(2)	0.904(1)
**11**	987(2)±40	1.148(3)±0.168	870(3)	0.903(1)
**12**	968(2)±22	1.135(4)±0.163	850(3)	0.905(1)
**13**	943(3)±21	1.117(6)±0.145	833(4)	0.903(1)
**14**	915(3)±15	1.114(6)±0.167	820(5)	0.913(1)
**15**	855(3)±4	1.075(5)±0.159	796(6)	0.908(1)
**16**	827(5)±15	1.063(12)±0.177	766(3)	0.941(1)
**17**	793(5)±43	1.013(9)±0.180	746(5)	0.951(1)
**18**	813(6)±13	1.102(13)±0.091	715(6)	0.951(1)
**19**	778(6)±4	1.063(16)±0.125	677(7)	0.945(1)
**20**	730(7)±18	1.042(14)±0.158	649(8)	0.969(1)

Table S7. Phase memory time constants (T_m) and stretch parameters (c) for **4** from Hahn echo decays. Uncertainty from statistical fitting in parentheses; errors estimated from repeat measurements on different sample given as range. All measurements were performed at the EDFS maximum.

Frequency	X-Band		Q-Band	
**Temperature / K**	**T_m / ns**	c	**T_m / ns**	c
**3**	5224±224	1.218(4)±0.043	5181(10)	1.162(4)
**4**	5437±313	1.186(3)±0.043	5445(10)	1.207(4)
**5**	5463±217	1.335(5)±0.082	5577(10)	1.254(4)
**6**	5478±141	1.342(3)±0.059	5750(8)	1.335(4)
**7**	5409±129	1.338(5)±0.053	5917(8)	1.431(4)
**8**	5302±137	1.323(5)±0.056	5763(7)	1.408(4)
**9**	5211±112	1.318(6)±0.052	5621(8)	1.390(4)
**10**	5121±14	1.304(4)±0.031	5381(10)	1.354(5)
**11**	4813±81	1.281(7)±0.009	5110(9)	1.329(4)
**12**	4606±17	1.234(9)±0.019	4730(11)	1.263(5)
**13**	4370±37	1.203(9)±0.020	4376(14)	1.212(6)
**14**	4022±54	1.163(8)±0.017	3986(12)	1.145(5)
**15**	3720±54	1.121(7)±0.021	3650(13)	1.099(5)
**16**	3458±67	1.137(10)±0.056	3221(21)	1.052(9)
**17**	3226±124	1.089(16)±0.034	2982(18)	1.043(8)
**18**	2830±84	1.029(11)±0.020	2626(28)	0.988(13)
**19**	2636±143	1.014(2)±0.023	2389(30)	0.972(14)
**20**	2340±86	0.974(2)±0.020	2198(27)	0.984(14)

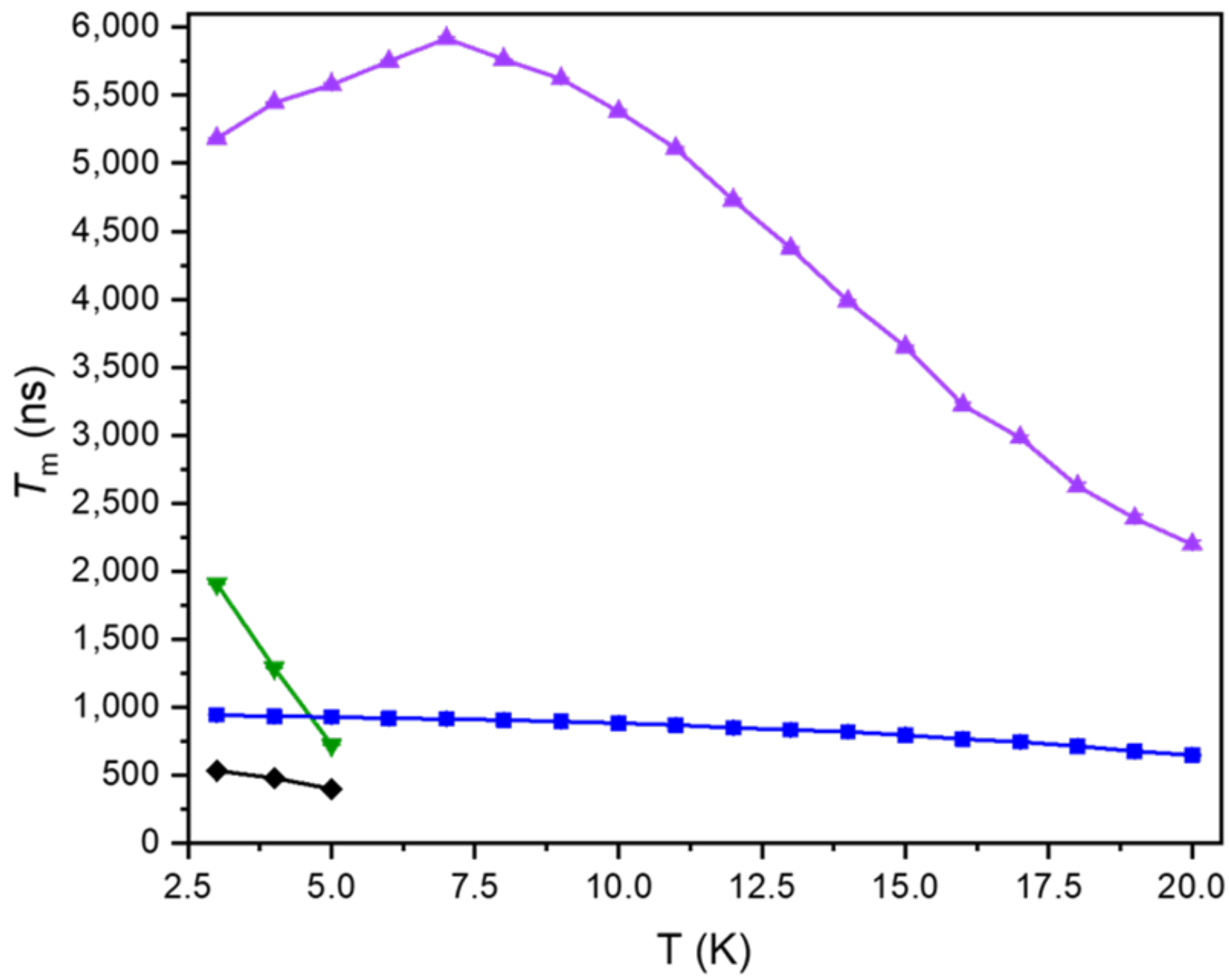

Figure S13. Phase memory time constant (T_m) measurement performed at Q-Band on 0.2 mM solutions for **1** (black line), **2** (green line), **3** (blue line) and **4** (purple line). Individual phase memory relaxation times are provided with experiment parameters in Tables S4, S5, S6 and S7.

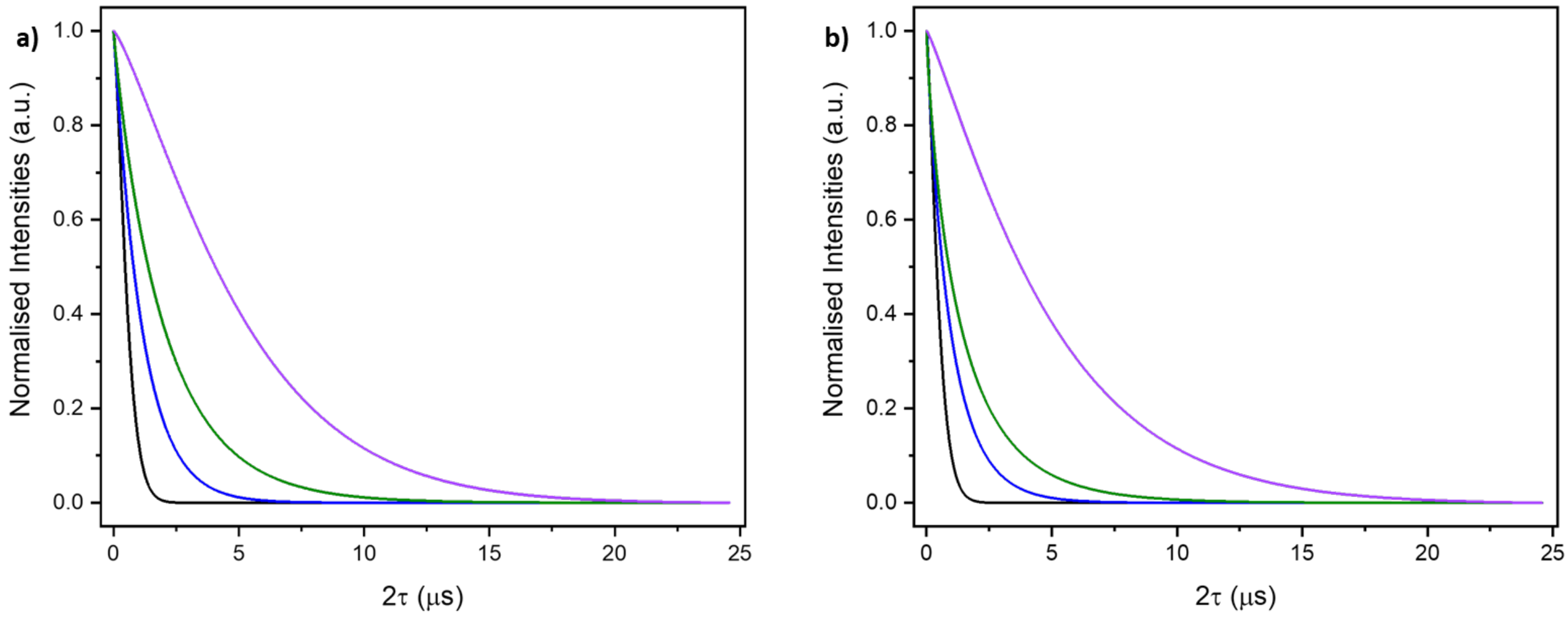

Figure S14. Comparison of phase memory time constants (T_m) measurements performed at 3 K for a) X-band and b) Q-band on 0.2 mM solutions in toluene for **1** (black line), **2** (green line), **3** (blue line) and **4** (purple line).

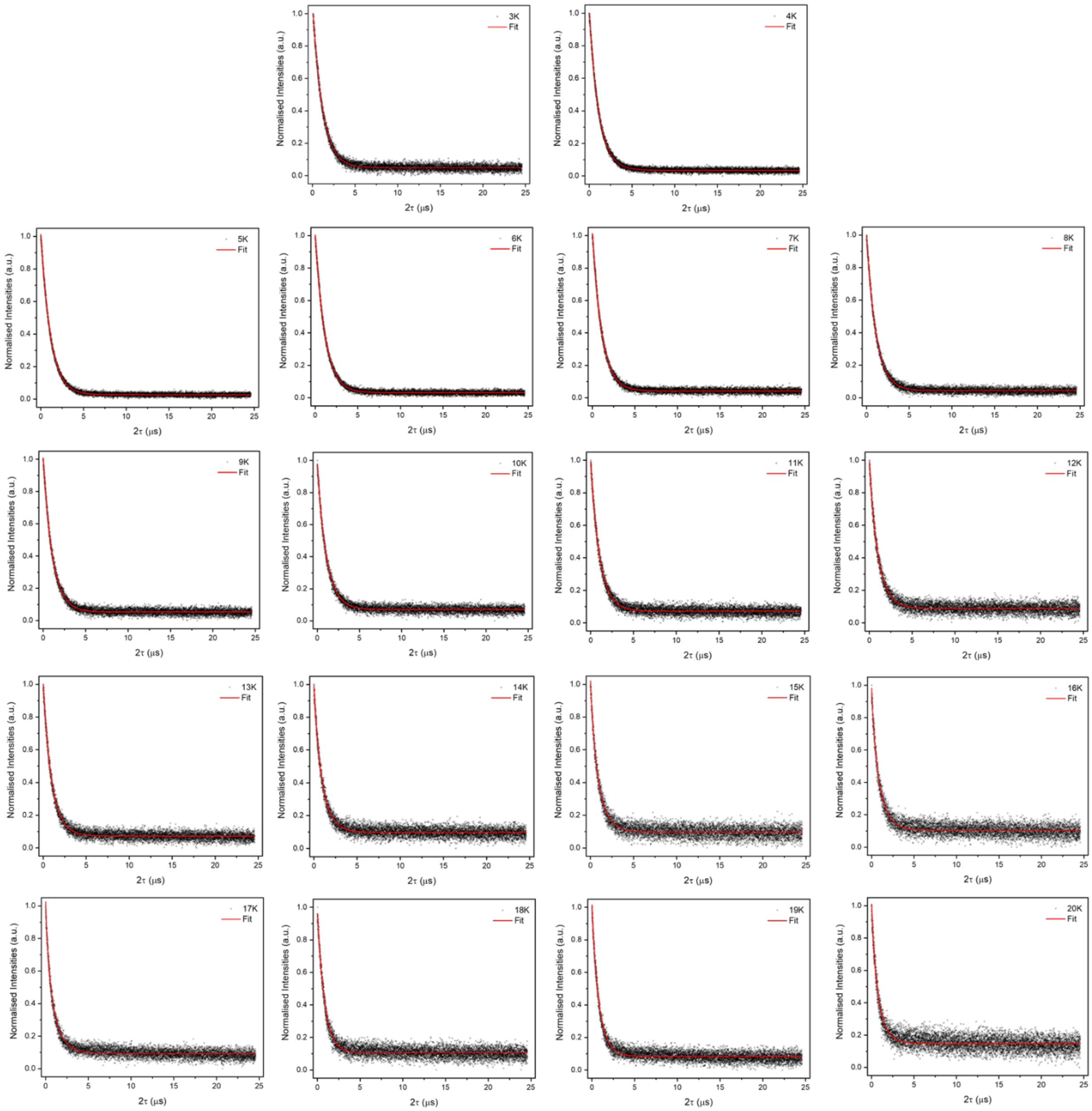

Figure S15. Phase memory time constants (T_m) measurements performed at X-band for **3**, on a 0.2 mM solution in toluene. Experimental data (black crosses) and fitting (red line).

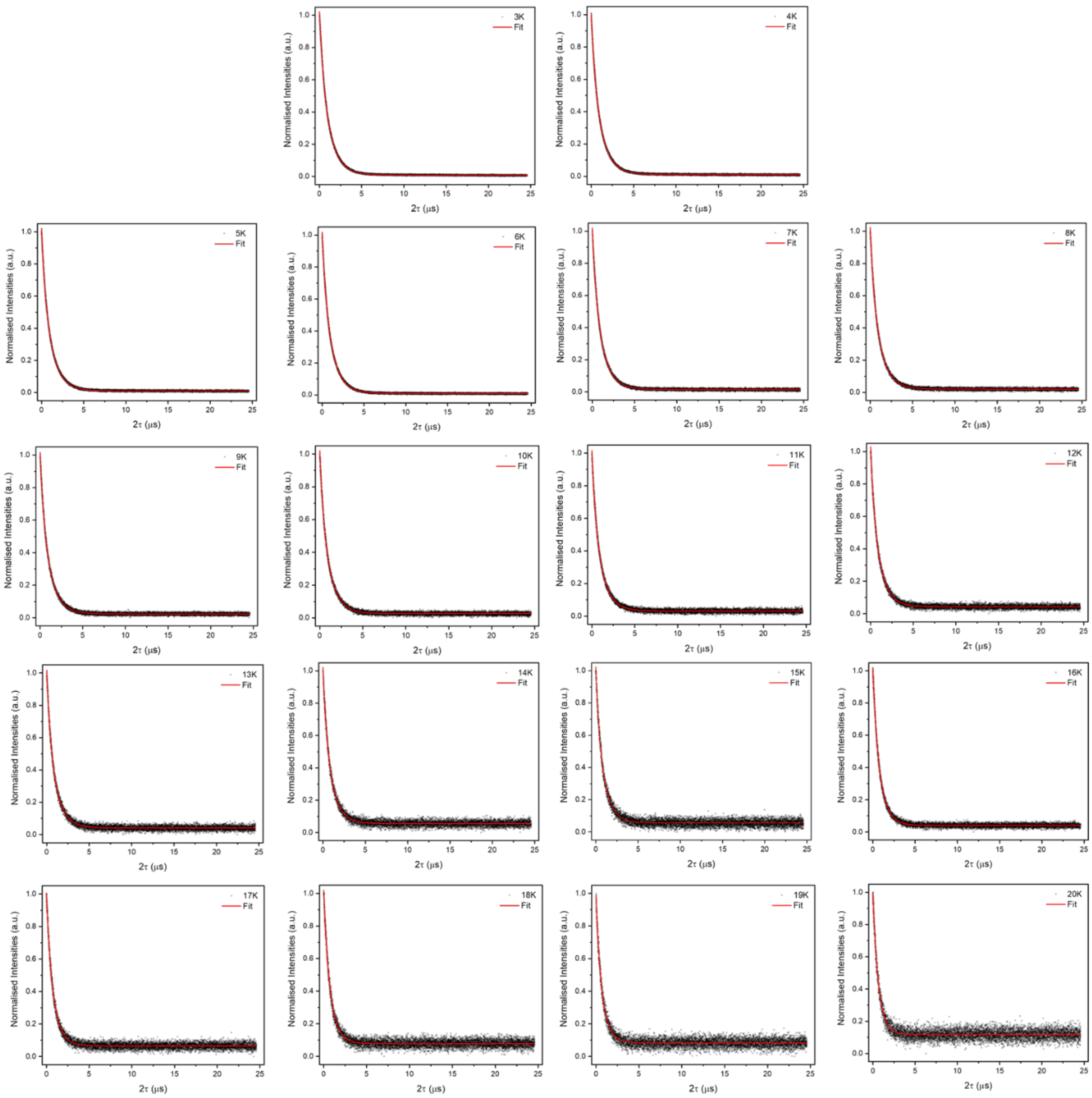

Figure S16. Phase memory time constant (T_m) measurements performed at Q-band for **3**, on a 0.2 mM solution in toluene. Experimental data (black crosses) and fitting (red line).

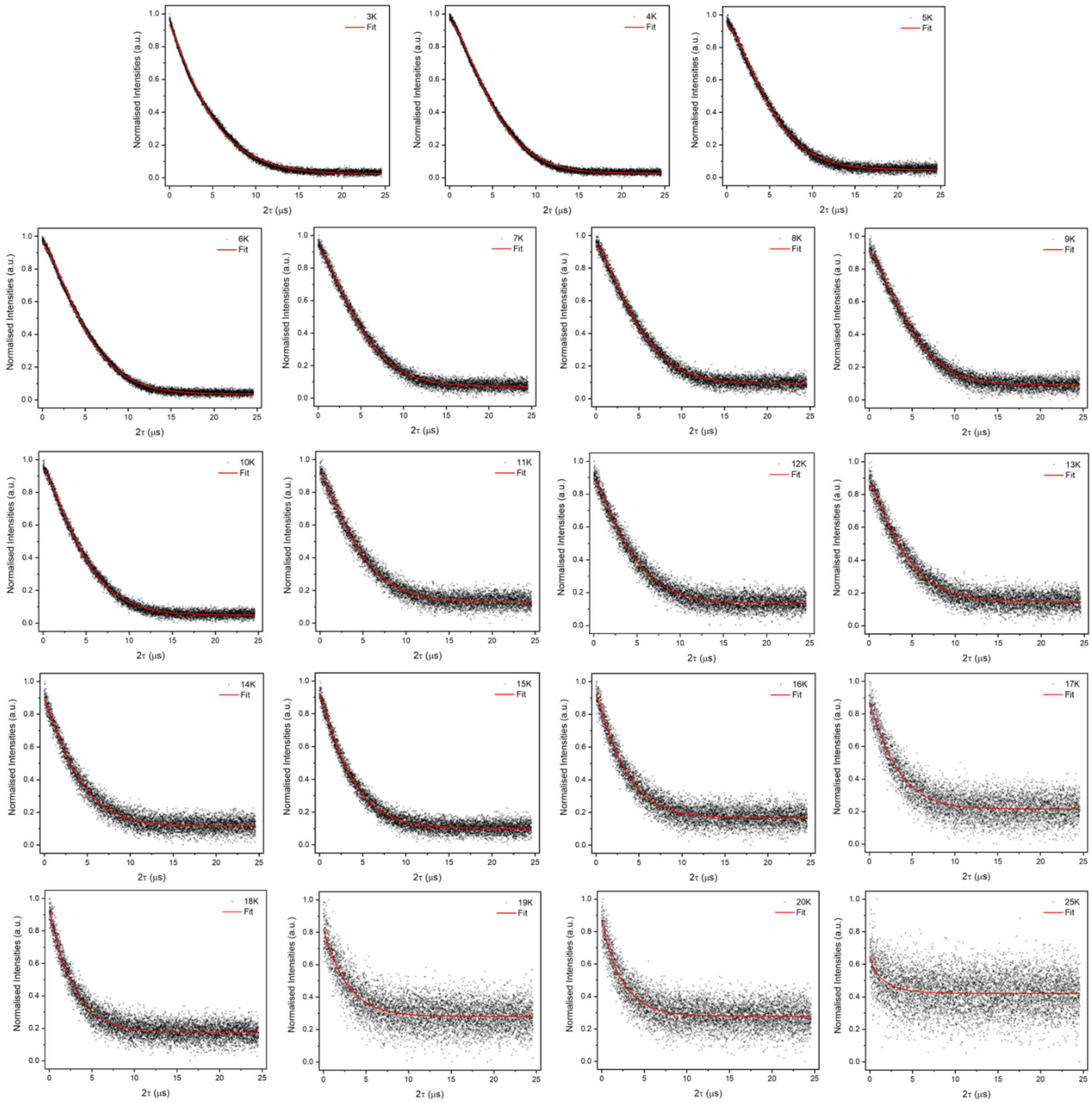

Figure S17. Phase memory time constant (T_m) measurements performed at X-band for **4**, on a 0.2 mM solution in toluene. Experimental data (black crosses) and fitting (red line).

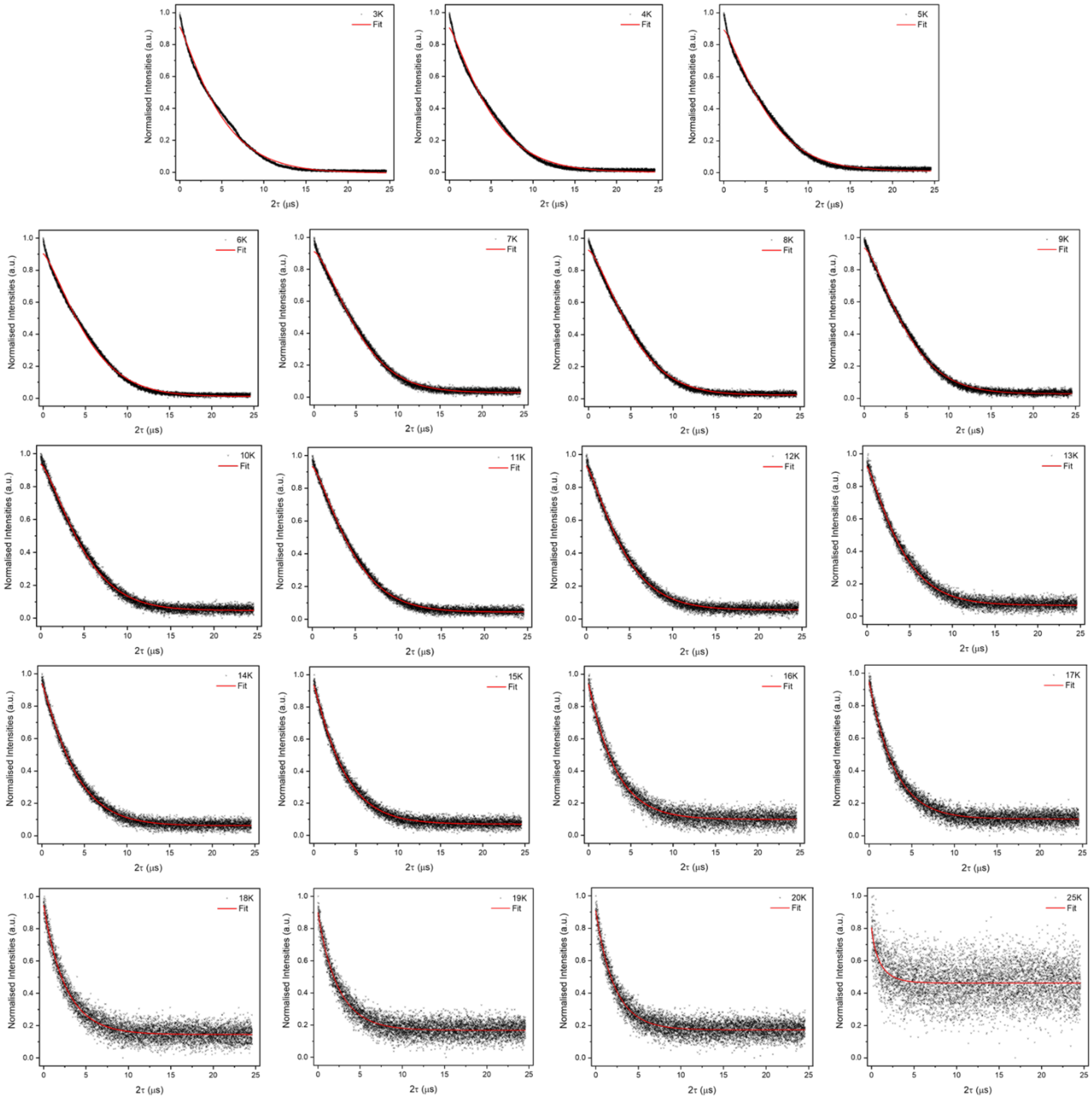

Figure S18. Phase memory time constant (T_m) measurements performed at Q-band for **4**, on a 0.2 mM solution in toluene. Experimental data (black crosses) and fitting (red line).

**Table S8**. Carr-Purcell-Meiboom-Gill (CMPG) time constants (T_{CMPG}) and stretch parameters (c) for **3** and **4** at X-band. Uncertainty from statistical fitting in parentheses. All measurements performed at EDFS maximum.				
**Compound**	**3**		**4**	
**Temperature / K**	T_{CMPG} **/ ns**	***c***	T_{CMPG} **/ ns**	***c***
**3**	5650(126)	0.324(3)	14481(225)	0.340(3)
**4**	5210(132)	0.301(3)	15442(233)	0.348(3)
**5**	4784(127)	0.291(3)	16638(255)	0.349(3)
**6**	4281(91)	0.303(3)	15620(246)	0.344(4)
**7**	4052(72)	0.324(3)	14271(208)	0.353(3)
**8**	3846(52)	0.356(3)	12469(168)	0.363(4)
**9**	3766(52)	0.385(3)	10805(133)	0.380(4)
**10**	2926(41)	0.388(3)	9434(105)	0.402(4)
**11**	2597(40)	0.409(4)	8074(85)	0.422(4)
**12**	2401(42)	0.440(5)	7154(70)	0.452(3)
**13**	2404(45)	0.479(6)	6366(68)	0.531(3)
**14**	2298(48)	0.504(7)	5870(72)	0.531(5)
**15**	2140(52)	0.540(10)	4984(64)	0.563(6)
**16**	2097(56)	0.559(12)	4526(60)	0.610(7)
**17**	1992(65)	0.607(17)	4322(66)	0.676(10)
**18**	1882(61)	0.680(22)	4255(69)	0.772(14)
**19**	1829(77)	0.664(27)	4116(77)	0.865(22)
**20**	1761(86)	0.715(37)	4076(78)	1.000(32)

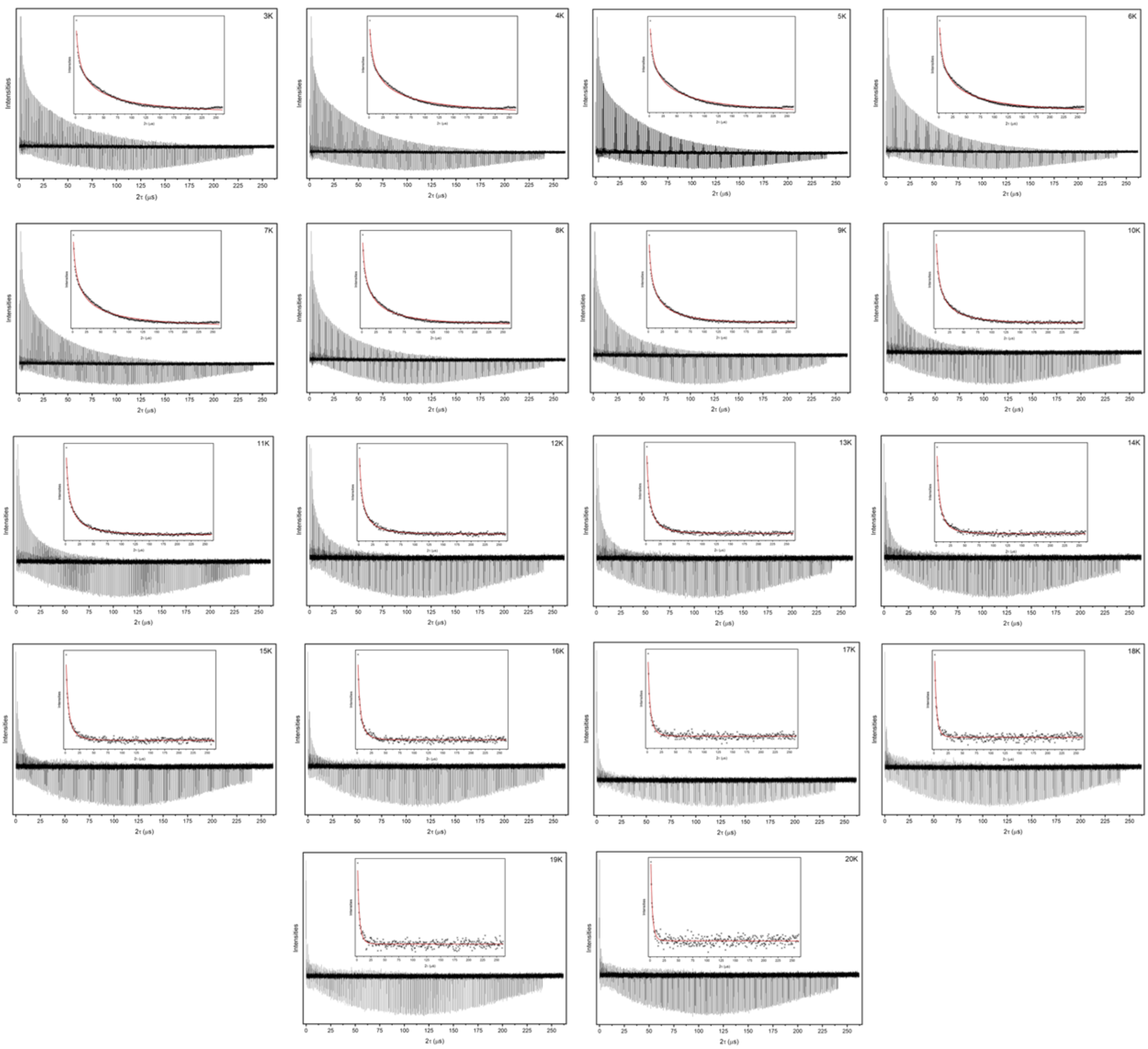

Figure S19. Carr-Purcell-Meiboom-Gill **(**CMPG) measurements (3-20 K) performed at X-band for **3**, on a 0.2 mM solution. Temperature given in figures. Raw data, with insert showing fitting of extracted peaks, experimental data (black crosses) and fitting (red line). Experiments used 20 and 40 ns pulses for π/2 and π, respectively, and a tau of 400 ns. 300 additional π refocusing pulses were used, following a standard Hahn echo detection sequence.

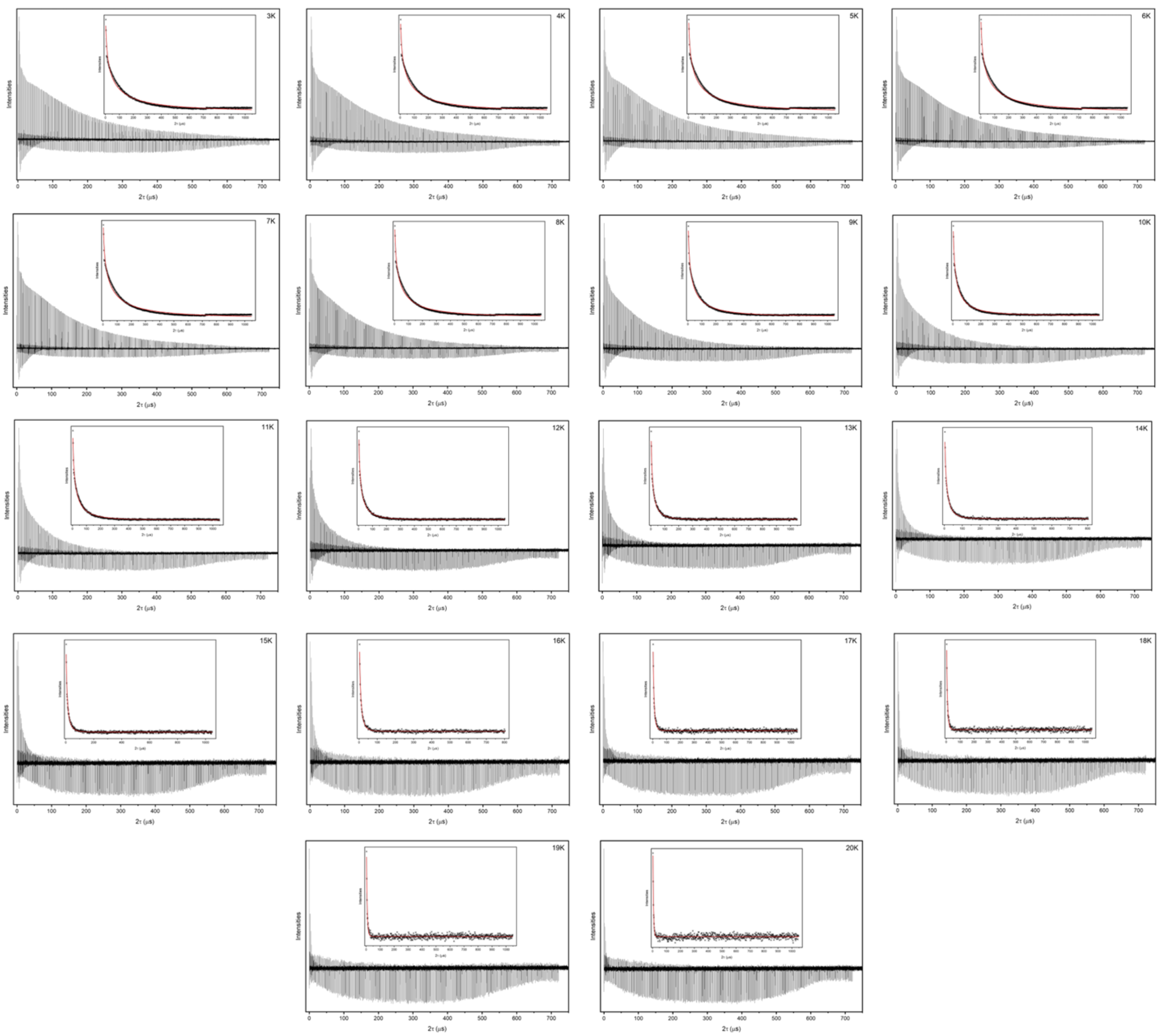

Figure S20. Carr-Purcell-Meiboom-Gill (CMPG) measurements (3-20 K) performed at X-band for **4**, on a 0.2 mM solution. Temperature given in figures. Raw data, with insert showing fitting of extracted peaks, experimental data (black crosses) and fitting (red line). Experiments used 20 and 40 ns pulses for π/2 and π, respectively, and a tau of 800 ns. 450 additional π refocusing pulses were used, following a standard Hahn echo detection sequence.

5.2.3 Spin Lattice Relaxation Times (T_1):

Spin lattice relaxation time constant (T_1) measurements were performed using a π*-*T*-π/2-*τ*-π-*τ*-echo sequence. All pulse, *T* and *τ* lengths are given below. The echo recovery was fit to an bi-exponential decay with the form $I(T) = I_1(T_0)\exp(-T/T_1) + I_2(T_0)\exp(-T/T_{SD})$, accounting for T_1 and spectral diffusion (SD), unless otherwise stated.

Table S9. Spin lattice relaxation time constant measurement parameters for compounds **1 - 4**:

Compound	1	2		3	4	1	1	2	3		4
Temperature (K)	3-4	3-4	5	3-20	3-20	3-4	5	3-5	3-10	11-20	3-20
π/2 pulse (ns)	40	40	40	20	20	20	20	20	20	20	20
π pulse (ns)	80	80	80	40	40	40	40	40	40	40	40
Inversion pulse π* (ns)	100	100	100	50	50	50	50	50	50	50	50
T (ns)	800	1000	500	1000	1000	1000	800	1000	1000	500	1000
τ (ns)	800	1000	500	1000	1000	1000	800	1000	800	500	1000

Table S10. Spin lattice relaxation time constants (T_1) for **1** and **2.**[23] Uncertainty from statistical fitting in parentheses; errors estimated from repeat measurements on different sample given as range. All measurements were performed at the EDFS maximum.

Compound	1		2	
	T_1 / μs		T_1 / μs	
Temperature / K	X-Band	Q-Band	X-Band	Q-Band
3	24(0.5)±3	28(1)	54(2)±3	61(1)
4	5(0.5)±0.5	6(0.5)	8(1)±1	11(0.5)
5	3(1)±1	2(0.5)	2(1)±1	3(0.5)

Table S11. Spin lattice relaxation time constants for **3.** Uncertainty from statistical fitting in parentheses; errors estimated from repeat measurements on different sample given as range. All measurements were performed at the EDFS maximum.

**Frequency**	**X-Band**		**Q-Band**	
**Temperature / K**	***T*₁ / *μ*s**	***T*SD / *μ*s**	***T*₁ / *μ*s**	***T*SD / *μ*s**
**3**	2718(182)±37	182(20)±15	2948(31)	217(5)
**4**	3048(205)±113	232(21)±3	1734(11)	195(2)
**5**	1997(128)±184	214(17)±55	1181(5)	157(1)
**6**	1275(41)±225	167(8)±25	829(4)	115(1)
**7**	854(48)±239	154(10)±1	568(3)	76(1)
**8**	488(15)±126	75(5)±7	364(2)	49(1)
**9**	739(14)±326	100(3)±45	241(2)	33(0.5)
**10**	497(15)±256	73(3)±39	147(1)	21(0.5)
**11**	341(12)±188	44(2)±25	97(1)	14(0.5)
**12**	200(8)±119	24(1)±15	72(1)	11(0.5)
**13**	157(10)±90	17(1)±10	48(1)	8(0.5)
**14**	105(11)±62	13(2)±7	35(1)	6(0.5)
**15**	108(27)±30	12(2)±4	30(1)	5(0.5)
**16**	56(9)±41	6(1)±5	24(1)	5(0.5)
**17**	40(10)±22	6(3)±3	21(1)	4(0.5)
**18**	29(14)±15	5(1)±1	18(1)	3(0.5)
**19**	18(8)±13	2(1)±0.6	15(1)	3(0.5)
**20**	15(9)±11	1(1)±0.7	6(1)	1(0.5)

Table S12. Spin lattice relaxation time constants for **4.** Uncertainty from statistical fitting in parentheses; errors estimated from repeat measurements on different sample given as range. All measurements were performed at the EDFS maximum.

**Frequency**	**X-Band**		**Q-Band**	
**Temperature / K**	***T*₁ / *μ*s**	***T*SD / *μ*s**	***T*₁ / *μ*s**	***T*SD / *μ*s**
**3**	2806(79)±51	243(9)±26	4500(15)	345(4)
**4**	2973(102)±38	194(11)±7	2300(18)	197(3)
**5**	2140(72)±42	206(10)±10	1600(10)	182(2)
**6**	1398(24)±102	185(5)±6	1160(5)	159(2)
**7**	987(28)±105	173(6)±11	654(5)	110(2)
**8**	609(10)±4	149(3)±30	423(3)	79(1)
**9**	415(10)±2	107(3)±22	282(3)	54(1)
**10**	254(8)±13	85(2)±34	175(2)	34(1)
**11**	161(7)±8	42(1)±9	132(3)	26(1)
**12**	106(6)±25	30(1)±4	74(2)	16(0.5)
**13**	75(6)±8	20(1)±2	61(2)	11(1)
**14**	56(5)±13	14(1)±3	41(2)	10(1)
**15**	58(5)±21	10(1)±2	35(2)	9(1)
**16**	22(4)±7	7(1)±1	23(1)	6(1)
**17**	30(8)±12	7(1)±2	20(0.5)	6(1)
**18**	31(9)±17	5(1)±1	17(1)	4(0.5)
**19**	7(3)±2	3(2)±1	16(1)	2(1)
**20**	12(7)±8	3(2)±1	11(2)	2(1)

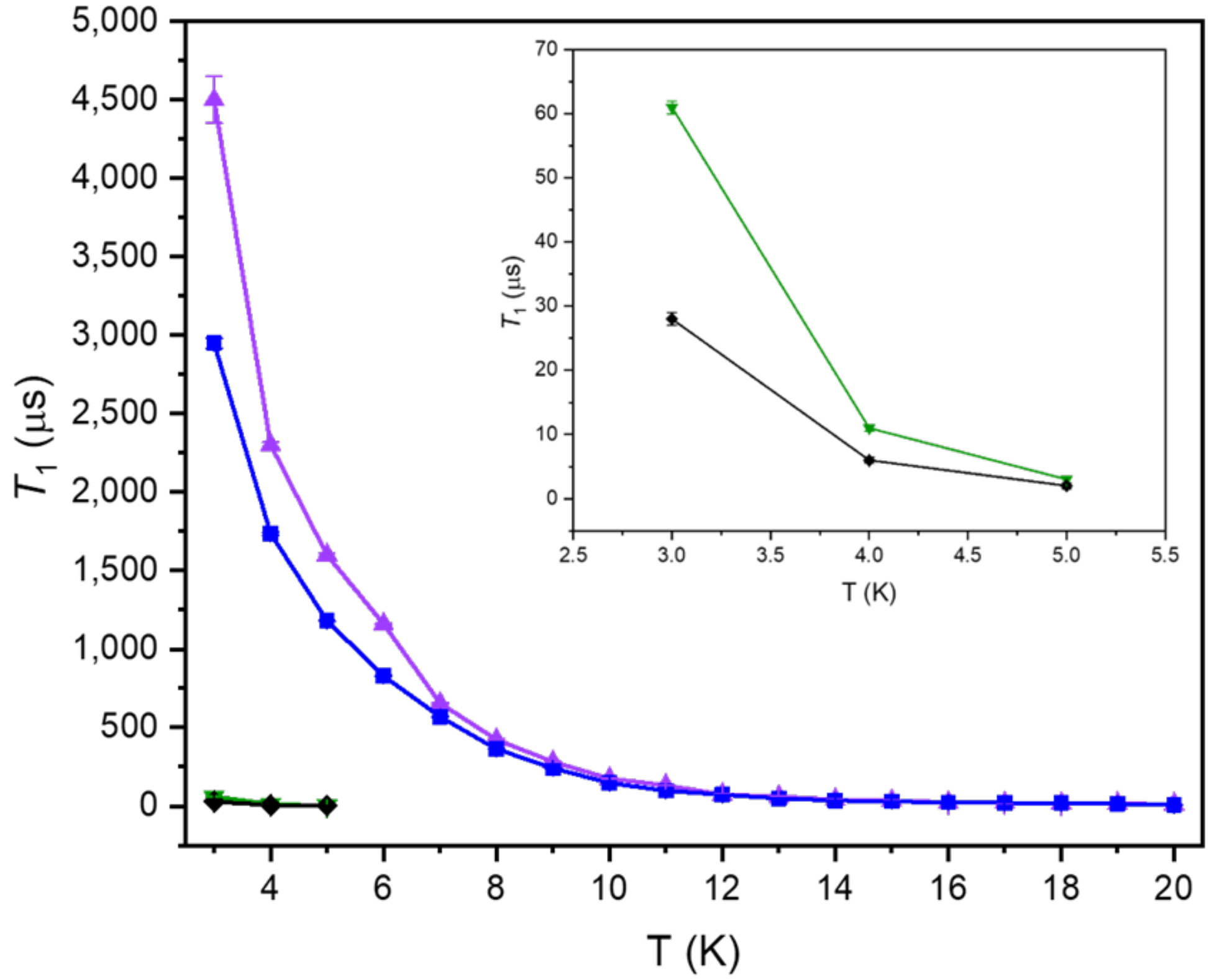

Figure S21. Spin lattice relaxation (T_1) measurements performed at Q-band on 0.2 mM solutions of **1** (black line), **2** (green line), **3** (blue line) and **4** (purple line). Individual phase memory relaxation times are provided with experiment parameters in Tables S9, S10, S11 and S12.

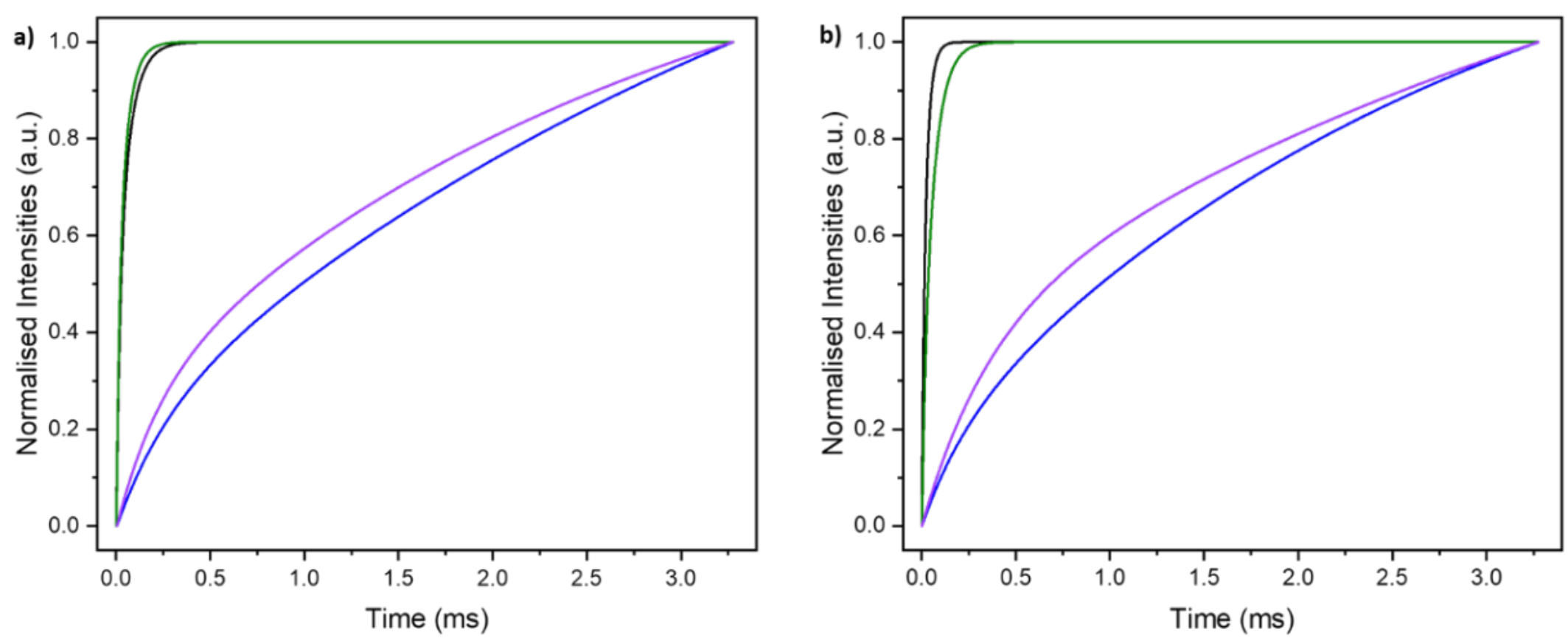

Figure S22. Comparison of spin lattice relaxation (T_1) measurements performed at 3 K for a) X-band and b) Q-band on 0.2 mM solutions for **1** (black line), **2** (green line), **3** (blue line) and **4** (purple line).

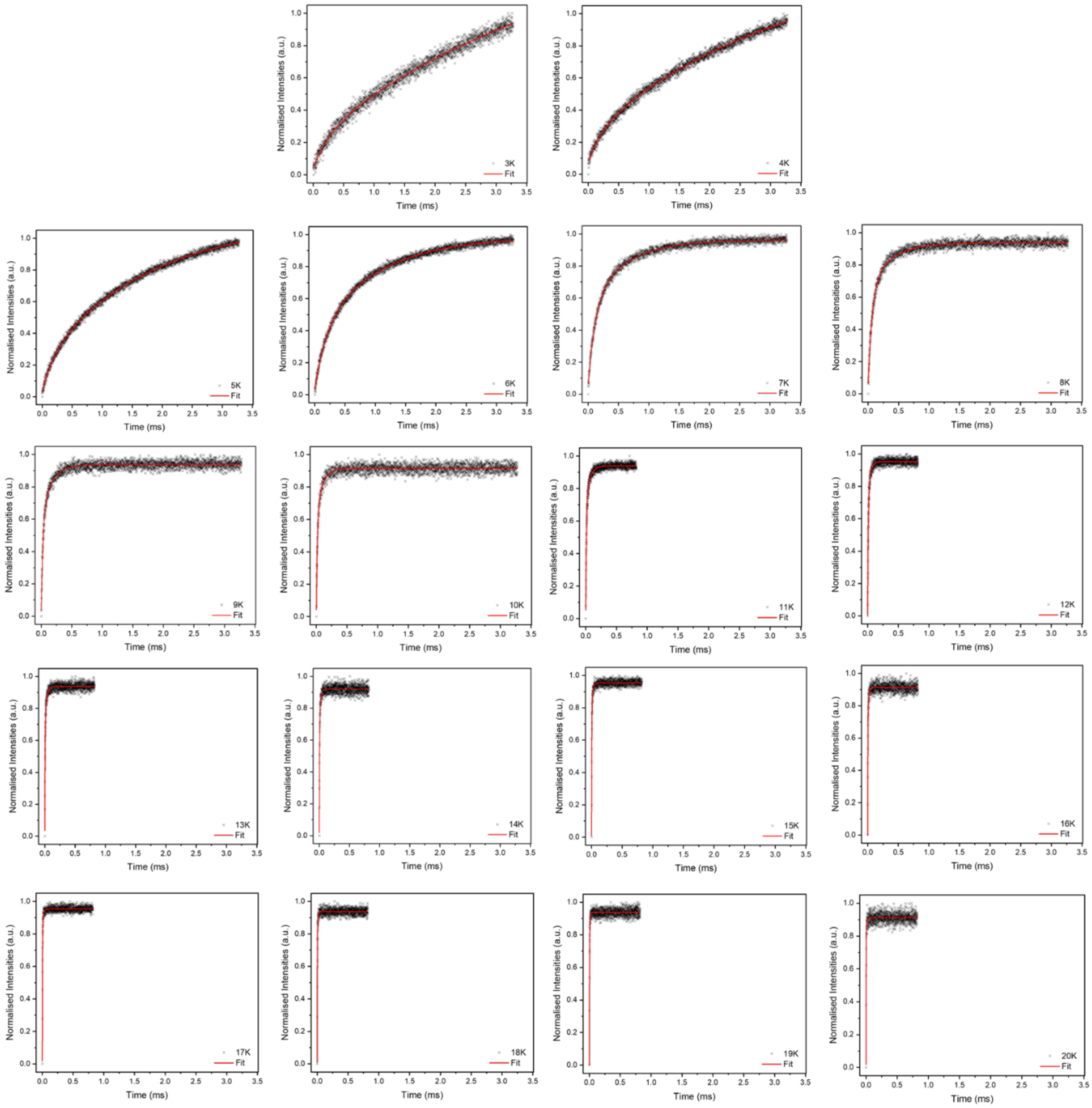

Figure S23. Spin lattice relaxation (T_1) measurements performed at X-band for **3**, on a 0.2 mM solution. Experimental data (black crosses) and fitting (red line).

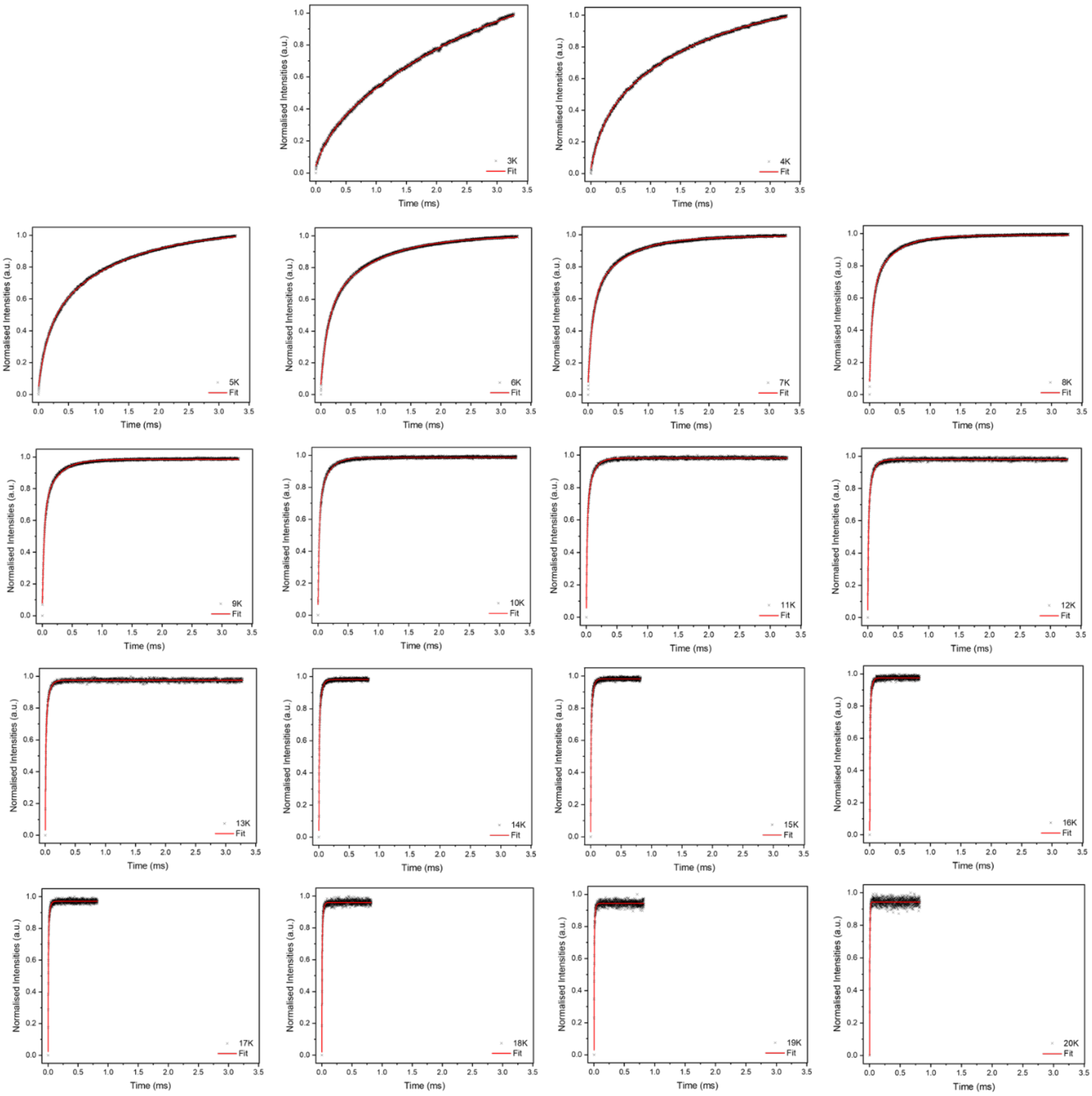

Figure S24. Spin lattice relaxation (T_1) measurements performed at Q-band for **3**, on a 0.2 mM solution. Experimental data (black crosses) and fitting (red line).

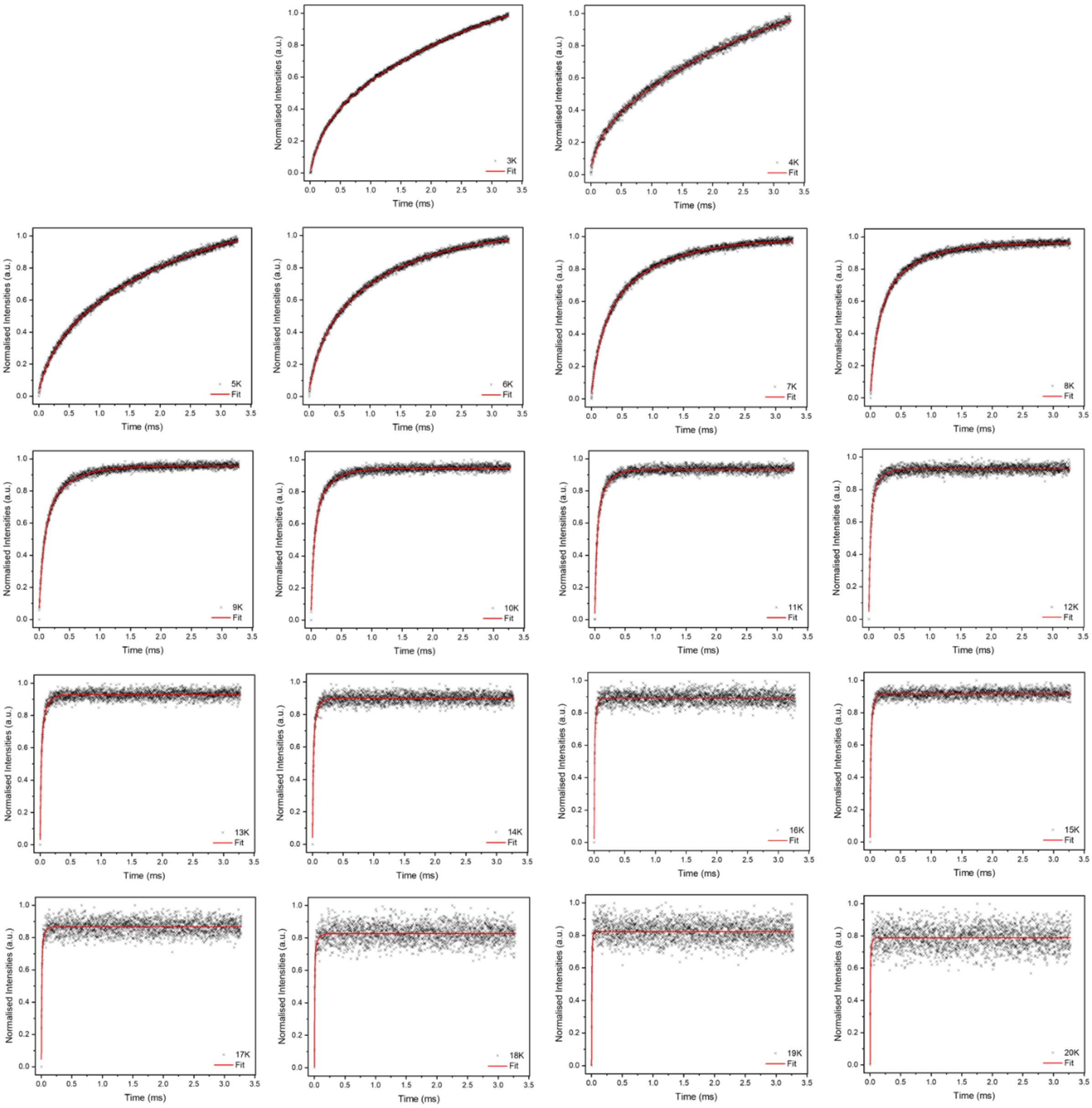

Figure S25. Spin lattice relaxation (T_1) measurements performed at X-band for **4**, on a 0.2 mM solution. Experimental data (black crosses) and fitting (red line).

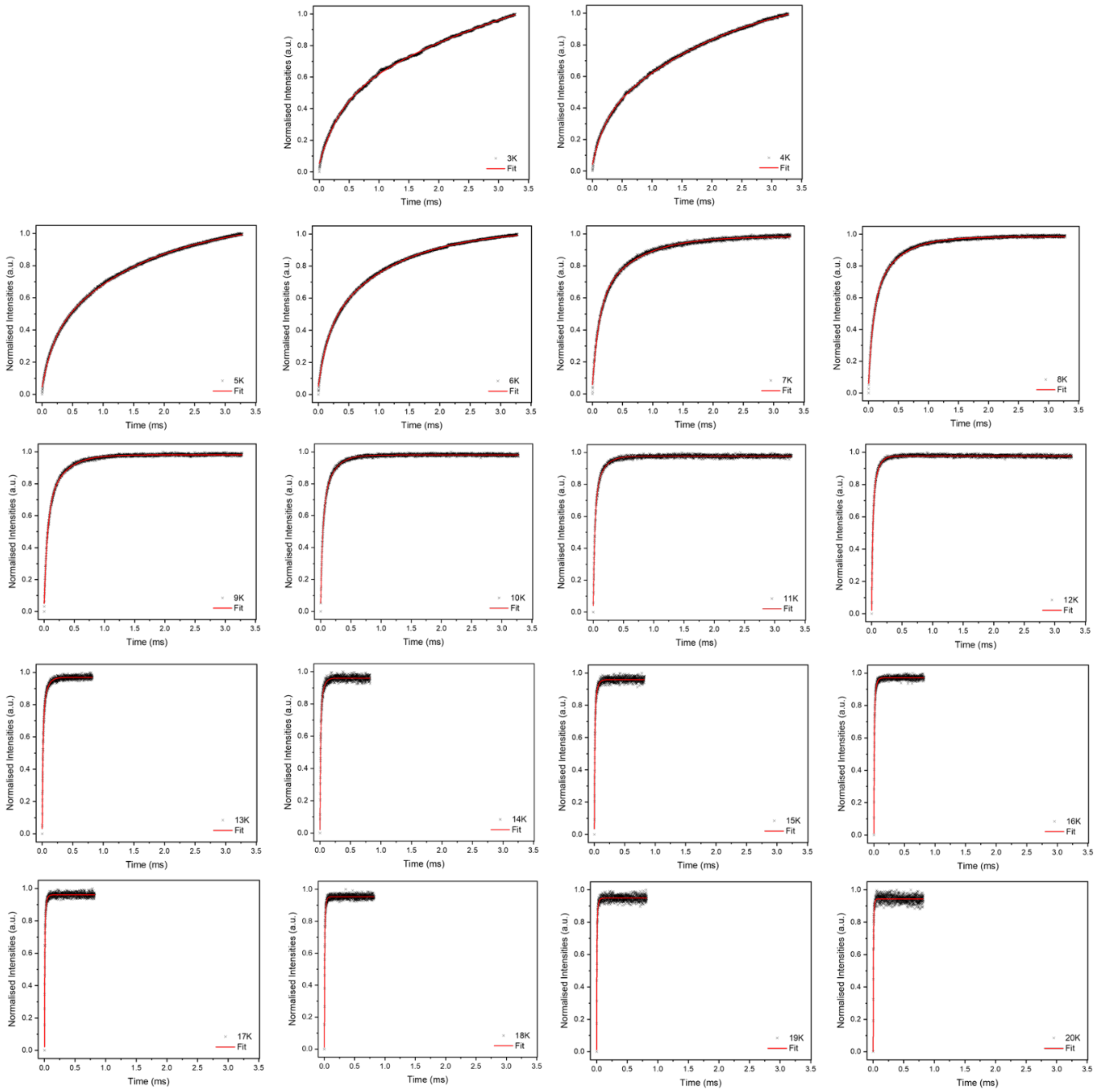

Figure S26. Spin lattice relaxation (T_1) measurements performed at Q-band for **4**, on a 0.2 mM solution. Experimental data (black crosses) and fitting (red line).

5.2.4. Transient nutation experiments:

All transient nutation measurements were performed using a π*-τ_1-π/2-τ_2-π pulse sequence with an initial π* pulse of 2 ns, increasing in increments of 2 ns. τ_1 and τ_2 were keep constant at 6,000 ns and 300 ns, respectively. The π/2 and π pulse lengths are detailed within each figures caption.

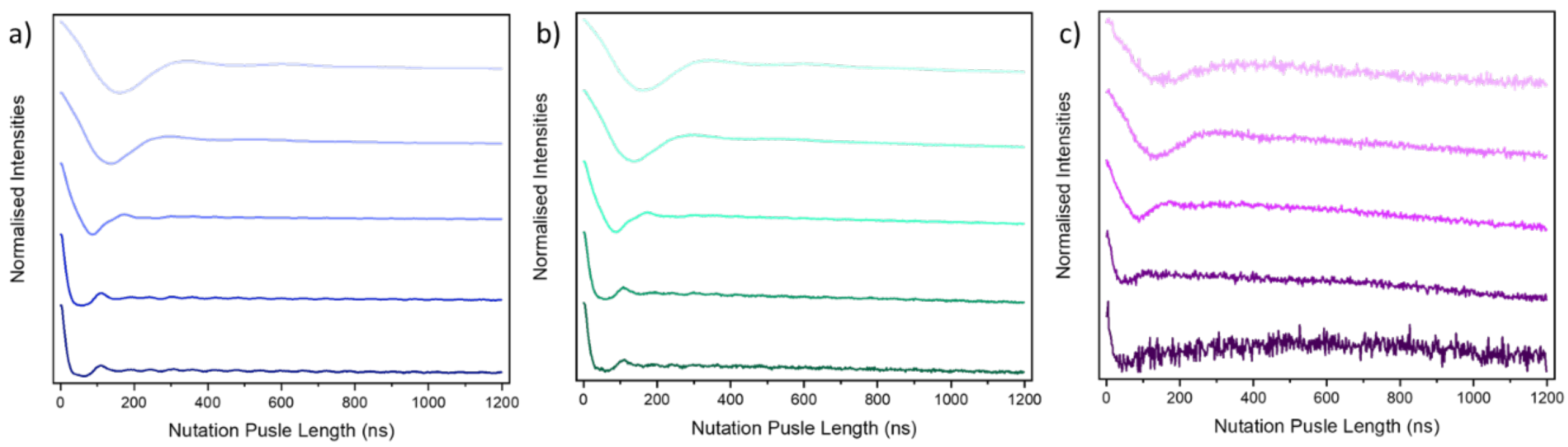

Figure S27. Rabi oscillations performed at X-band for **1**, on a 0.2 mM solution at a) 3 K, b) 4 K and c) 5 K. Pulse lengths for π/2 and π pulses are 10 ns and 20 ns, 20 ns and 40 ns, 40 ns and 80 ns, 60 ns and 120, and 80 ns and 160 ns, from dark to light colour lines, respectively.

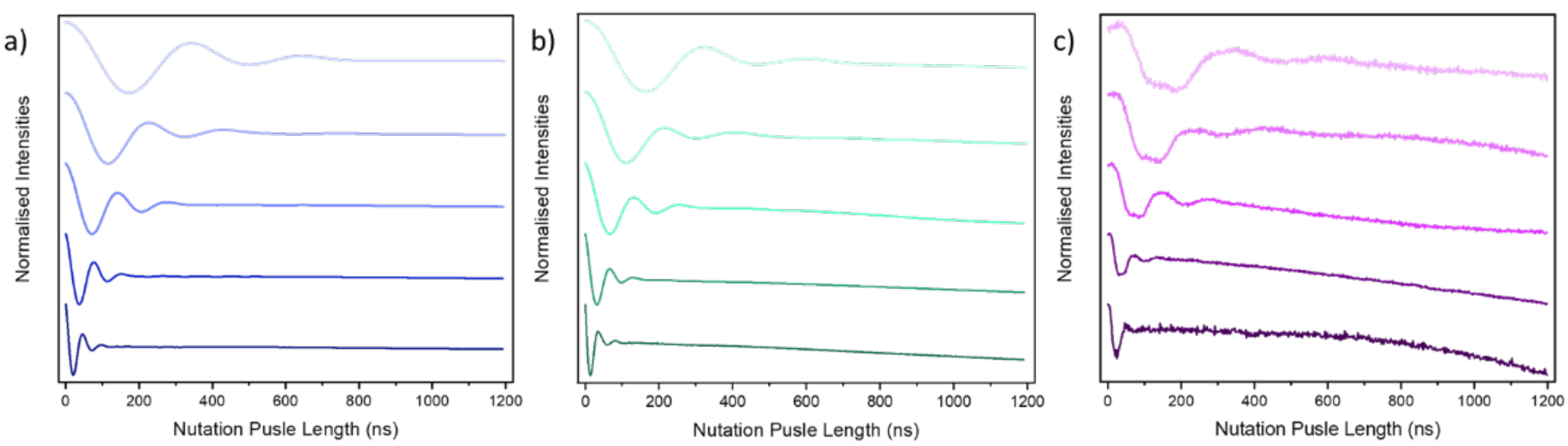

Figure S28. Rabi oscillations performed at Q-band for **1**, on a 0.2 mM solution at a) 3 K, b) 4 K and c) 5 K. Pulse lengths for π/2 and π pulses are 10 ns and 20 ns, 20 ns and 40 ns, 40 ns and 80 ns, 60 ns and 120, and 80 ns and 160 ns, from dark to light colour lines, respectively.

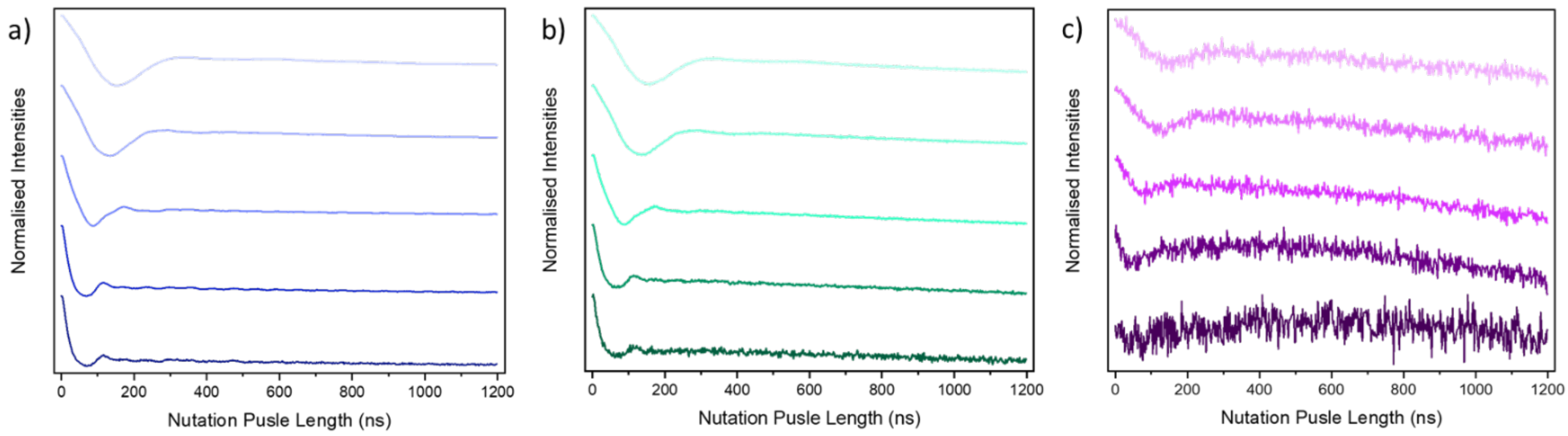

Figure S29. Rabi oscillations performed at X-band for **2**, on a 0.2 mM solution at a) 3 K, b) 4 K and c) 5 K. Pulse lengths for π/2 and π pulses are 10 ns and 20 ns, 20 ns and 40 ns, 40 ns and 80 ns, 60 ns and 120, and 80 ns and 160 ns, from dark to light colour lines, respectively.

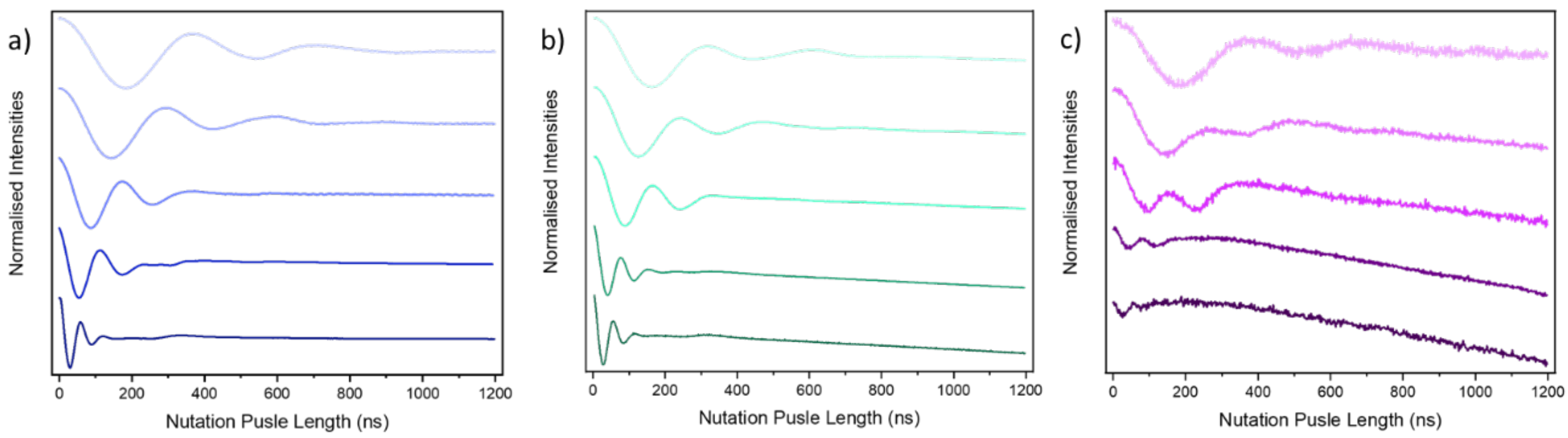

Figure S30. Rai oscillations performed at Q-band for **2**, on a 0.2 mM solution at a) 3 K, b) 4 K and c) 5 K. Pulse lengths for π/2 and π pulses are 10 ns and 20 ns, 20 ns and 40 ns, 40 ns and 80 ns, 60 ns and 120, and 80 ns and 160 ns, from dark to light colour lines, respectively.

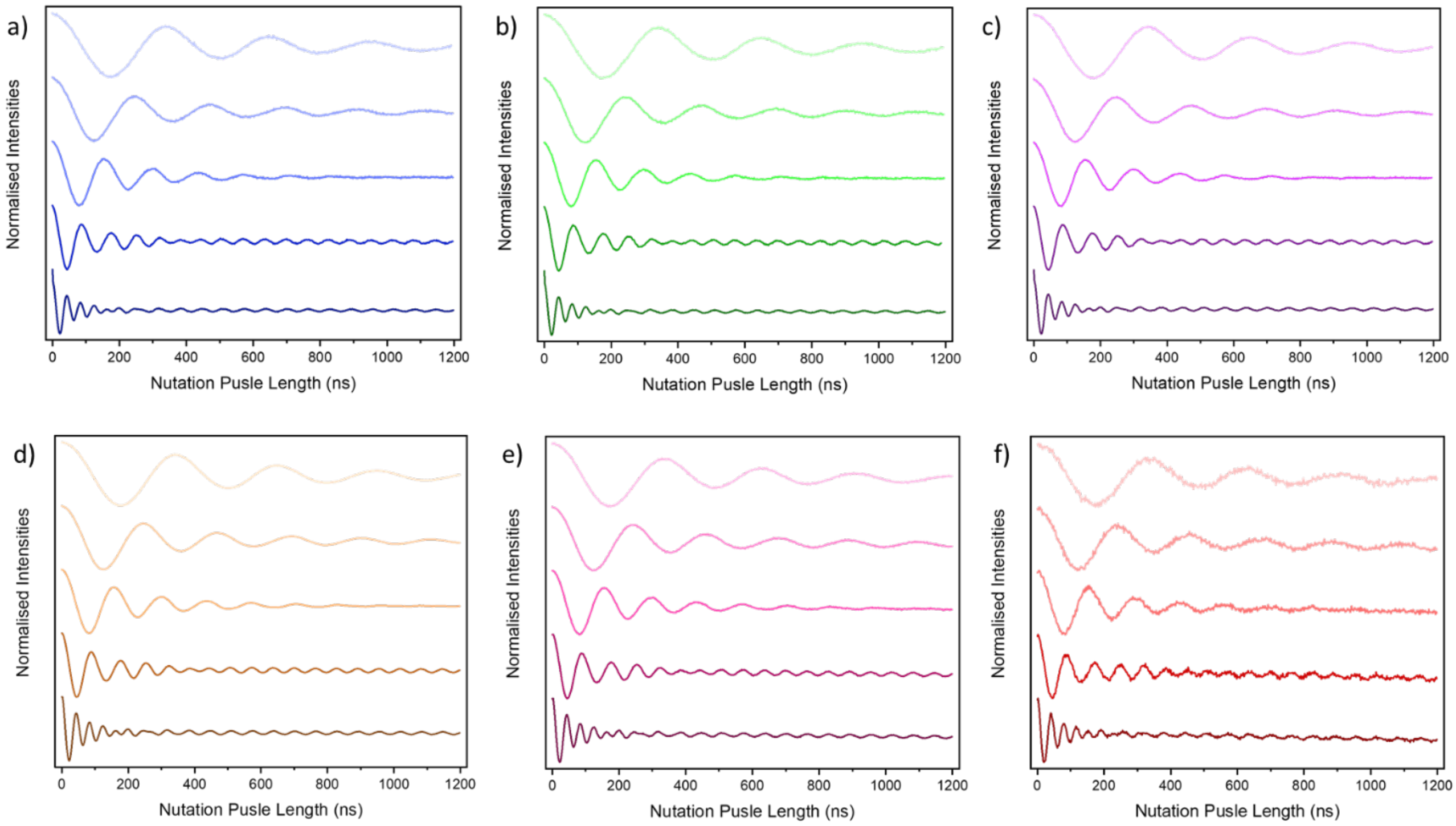

Figure S31. Rabi oscillations performed at X-band for **3**, on a 0.2 mM solution at a) 3 K, b) 4 K, c) 5 K, d) 10 K, e) 15 K and f) 20 K. Pulse lengths for π/2 and π pulses are 10 ns and 20 ns, 20 ns and 40 ns, 40 ns and 80 ns, 60 ns and 120, and 80 ns and 160 ns, from dark to light colour lines, respectively.

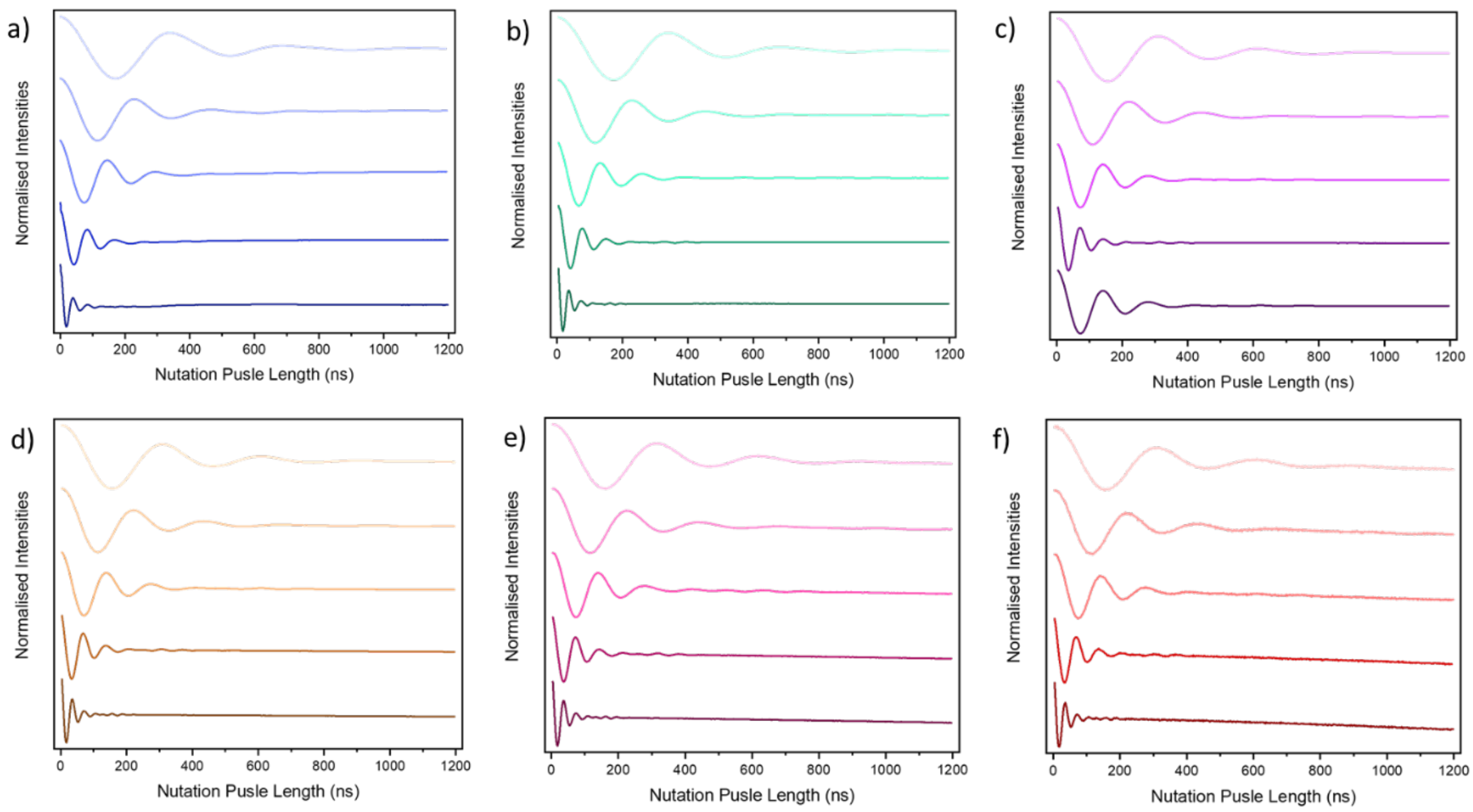

Figure S32. Rabi oscillations performed at Q-band for **3**, on a 0.2 mM solution at a) 3 K, b) 4 K, c) 5 K, d) 10 K, e) 15 K and f) 20 K. Pulse lengths for π/2 and π pulses are 10 ns and 20 ns, 20 ns and 40 ns, 40 ns and 80 ns, 60 ns and 120, and 80 ns and 160 ns, from dark to light colour lines, respectively.

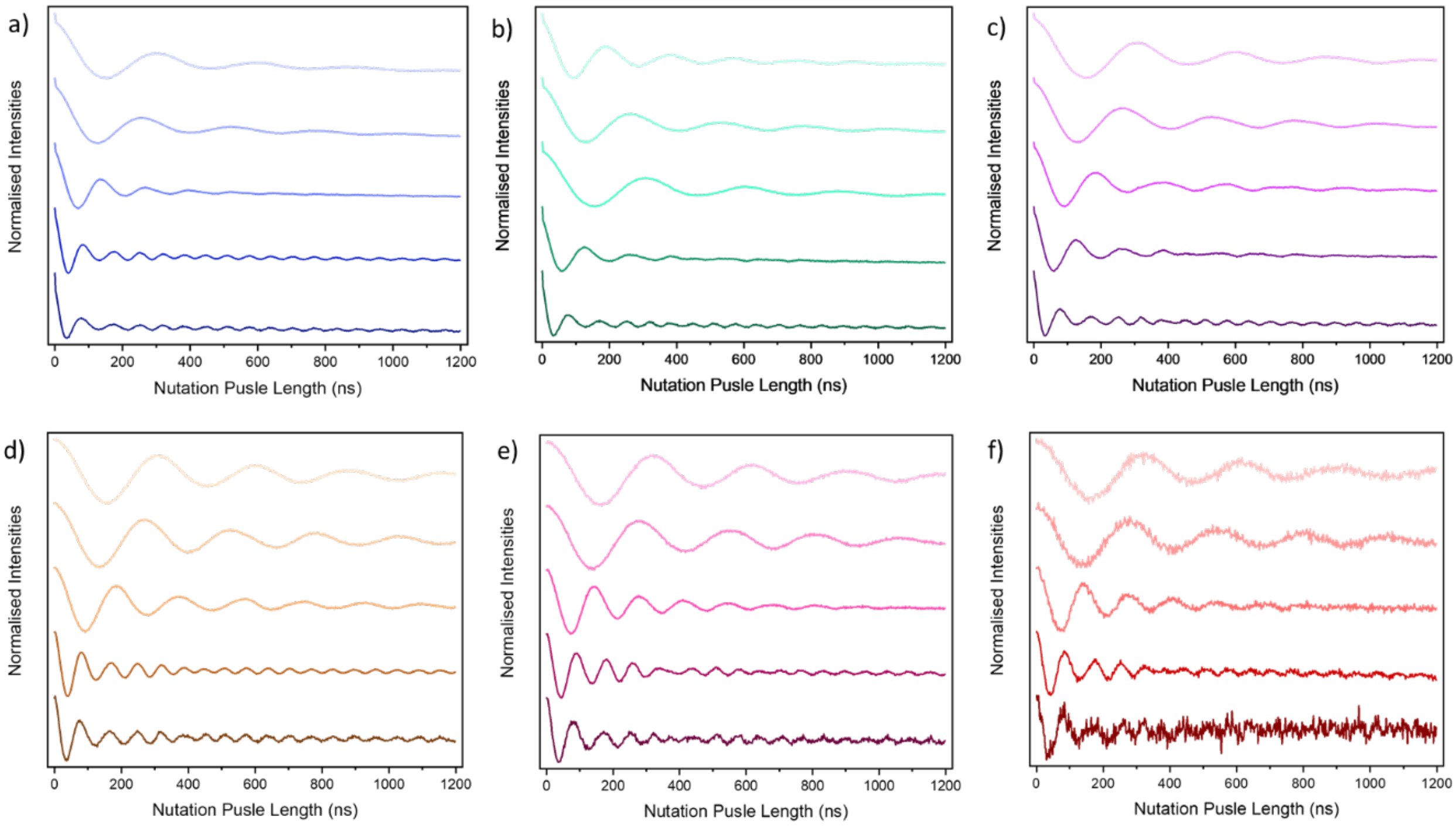

Figure S33. Rabi oscillations performed at X-band for **4**, on a 0.2 mM solution at a) 3 K, b) 4 K, c) 5 K, d) 10 K, e) 15 K and f) 20 K. Pulse lengths for π/2 and π pulses are 10 ns and 20 ns, 20 ns and 40 ns, 40 ns and 80 ns, 60 ns and 120, and 80 ns and 160 ns, from dark to light colour lines, respectively.

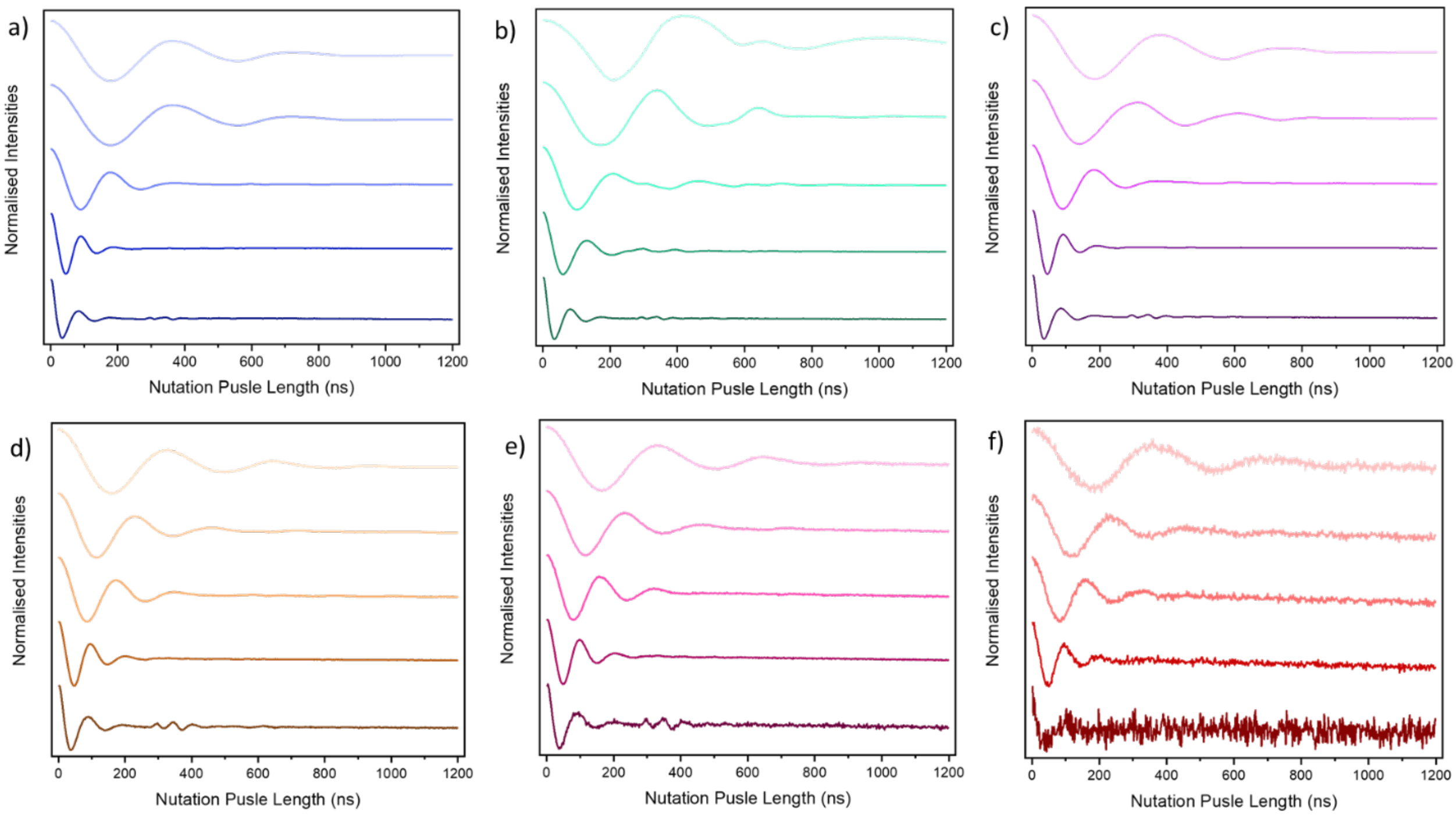

Figure S34. Rabi oscillations performed at Q-band for **4**, on a 0.2 mM solution at a) 3 K, b) 4 K, c) 5 K, d) 10 K, e) 15 K and f) 20 K. Pulse lengths for π/2 and π pulses are 10 ns and 20 ns, 20 ns and 40 ns, 40 ns and 80 ns, 60 ns and 120, and 80 ns and 160 ns, from dark to light colour lines, respectively.

6. References

S1. (a) Sheldrick, G. M. *SADABS*, empirical absorption correction program based upon the method of Blessing. (b) Krause, L.; Herbst-Irmer, R.; Sheldrick, G. M.; Stalke, D. An empirical correction for absorption anisotropy *J. Appl. Cryst.* **2015**, *48*. (c) Blessing, R. H. An empirical correction for absorption anisotropy, *Acta Crystallogr.* **1995**, *A51*, 33-38.

S2. (a) Sheldrick. G. M. Crystal structure refinement with SHELXL, *Acta Crystallogr.*, 2015, *C71*, 3-8; (b) Dolomanov, O. V.; Bourhis, L. J.; Gildea, R. J.; Howard, J. A. K.; Puschmann, H. OLEX2: a complete structure solution, refinement and analysis program. *J. Appl. Cryst*., **2009,** *42*, 339–341.

S3. Stoll, S.; Schweiger. A. EasySpin, a comprehensive software package for spectral simulation and analysis in EPR. *J. Magn. Reson*., **2006**, *178*, 42-55.
